# Land cover change across the major proglacial regions of the sub-Antarctic islands, Antarctic Peninsula, and McMurdo Dry Valleys, during the 21^st^ century

**DOI:** 10.1080/15230430.2025.2483474

**Published:** 2025-05-07

**Authors:** Christopher D. Stringer, Jonathan L. Carrivick, Duncan J. Quincey, Daniel Nývlt, Alexis Comber

**Affiliations:** a School of Built Environment, Engineering and Computing, Leeds Beckett University, Leeds, UK; b School of Geography and Water, University of Leeds, Leeds, UK; c Polar-Geo-Lab, Department of Geography, Faculty of Science, Masaryk University, Brno, Czech Republic

**Keywords:** Antarctica, proglacial, LULC, land cover, sediment

## Abstract

Land cover information is essential for understanding Earth surface processes and ecosystems. Here, we use *K*-means clustering to classify Landsat 8 Operational Land Imager (OLI) images covering six proglacial sites of sub-Antarctic islands, the Antarctic Peninsula, and the McMurdo Dry Valleys at 30-m resolution. We quantify spatial patterns of water, bedrock, vegetation, and sediments to an accuracy of 77 percent. Vegetation is most abundant on South Georgia (7 percent of the proglacial area) and the South Shetland Islands (1 to 2 percent). Furthermore, we use change vector analysis (CVA) to discriminate landcover change in the twenty-first century. A latitudinal pattern is evident in ice loss and proglacial landscape change; for example, loss of ice on South Georgia and proglacial landcover change is two orders of magnitude greater than in the McMurdo Dry Valleys. Four of the studied sites had similar landscape stability (64 to 68 percent unchanged), with Alexander Island an exception (50 percent change) due to recent enhanced glacier melt. Overall, we show how landcover of proglacial regions of the climaticallysensitive sub-Antarctic and Antarctica has changed since 2000, with a CVA accuracy of 80 percent. These findings inform understanding of geomorphological activity and sediment and nutrient fluxes and hence terrestrial and marine ecosystems.

## Introduction

Consistent land cover information is essential to furthering our understanding of terrestrial environments, ecological niches, and the atmosphere, especially across sensitive regions of Earth (Raup et al. 2007; Ban, Gong, and Giri 2015; Chen, Li, and Wang 2019; Gong et al. 2020). Additionally, land cover maps are a critical resource required to support the research of climate change, particularly those that include information on vegetation coverage (Bojinski et al. 2014). Different types of land cover can change or respond to climatic forcing in different ways, depending on their physical and chemical properties (GCOS 2010). Owing to the frequent return period and extensive areas covered by satellite images, land cover maps are increasingly being produced using remote sensing techniques and the changes occurring in the landscape can thus be detected and quantified (Friedl et al. 2010; Lea 2018; Brown et al. 2022). Several global land cover products have been released in recent years (e.g., Brown et al. 2022), but they typically do not include Antarctica or sub-Antarctic Islands (e.g., South Georgia), leaving a gap in our understanding of Earth’s southernmost continent.

**Figure 1.**
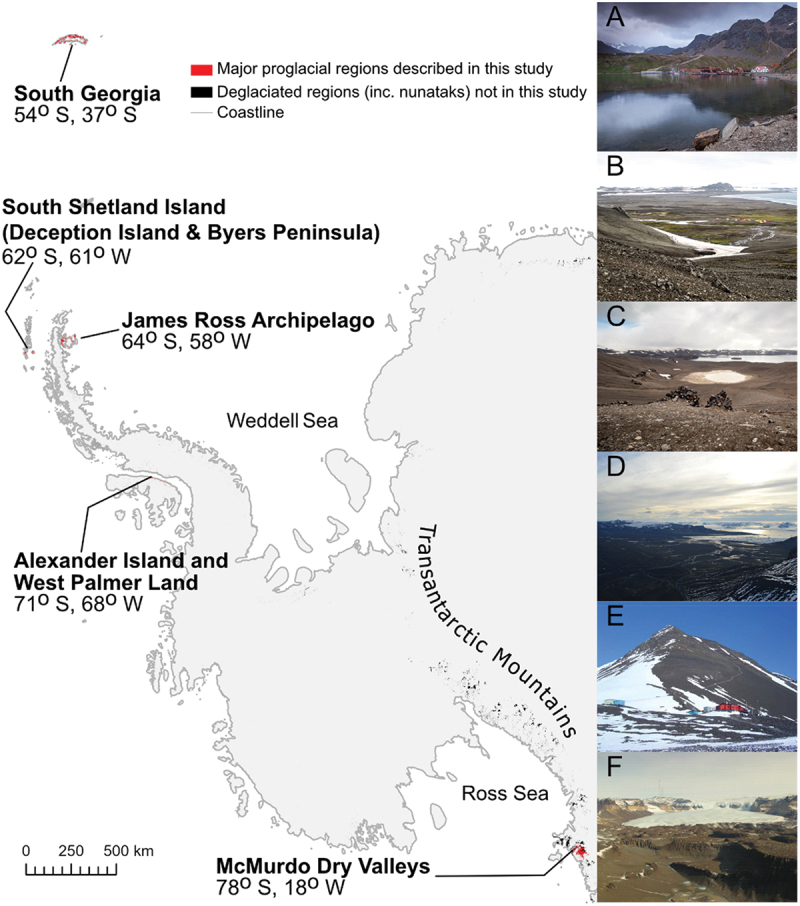
Location of our study sites. The areas analyzed are highlighted in red and span a latitudinal gradient from 54° S to78° S. Proglacial regions not analyzed in this study are highlighted in black (Burton-Johnson et al. 2016) and are primarily mountains (e.g., Transantarctic Mountains) or are frequently covered by extensive cloud cover (e.g., King George Island). (A) Grytviken on South Georgia. Taken in 2009 by Simon Murgatroyd (CC BY-SA 2.0). (B) Camp Byers on South Beach (ESP) on Byers Peninsula. Taken in 2017 by “Inoceramid bivalves” (CC BY-SA 4.0). (C) Telefon Bay (background), as viewed from the rim of a crater on Deception Island. Taken in 2020 by Espen Mills (CC BY-SA 4.0). (D) Abernethy Flats on James Ross Island’s Ulu Peninsula, as viewed from Lachman Crags, above Triangular Glacier (looking West), taken in 2022. (E) The central station of Fossil Bluff on Alexander Island in 2003. Photo taken in 2003 by “Apacheeng lead” (Public Domain). (F) The Wright Valley of the McMurdo Dry Valleys (looking west toward Wright Upper Glacier) in 2013, taken by “Turkish D.” (CC BY-SA 4.0). Inset photos (A), (B), (D), (E), and (F) were sourced from Wikimedia Commons. Photo (C) by Christopher D. Stringer.

The majority (99.8 percent) of Antarctica is covered by ice, with the remaining 0.2 percent characterized as nunataks (i.e., mountain peaks that penetrate the ice sheet) or as proglacial regions (Burton-Johnson et al. 2016; Figure 1). Proglacial regions are predominantly shaped by the interplay of meltwater from glaciers, which erodes, transports, and deposits sediment, and hillslope activity, which largely acts to supply new sediment into the system during mass transport events. In a warming climate, the activity of water and increased mass movements result in greater sediment discharge (Ballantyne 2008; Klaar et al. 2015; Staines et al. 2015). In polar regions, where permafrost can be extensive, the active layer is an additional and important water and sediment source on days when ground temperatures exceed 0°C (Humlum, Instanes, and Sollid 2003; Kavan et al. 2017; Costa et al. 2018; Łepkowska and Stachnik 2018). All of these factors mean that the Antarctic landscape is highly dynamic.

Maps of land cover and land cover change are particularly important for Antarctica, owing to its dynamic landscape and rapid environmental change (Davies et al. 2013). Unlike most other regions on Earth, human activities are not the major control on land cover type in Antarctica, and the footprint of anthropogenic activities is limited to relatively small areas (Tejedo et al. 2016, 2022). Until the start of the twenty-first century, the Antarctic Peninsula Region (APR) was one of the most rapidly warming places on Earth, with a temperature rise of 1.5°C observed since the 1950s (Vaughan et al. 2003; Mulvaney et al. 2012; Oliva et al. 2017). Following a hiatus in warming at the start of the twenty-first century, there is evidence that this trend has resumed (Carrasco, Bozkurt, and Cordero 2021) and glaciers have continued to respond to the temperature increases of the twentieth century and subsequent warming since 2015 (Oliva et al. 2017; Engel et al. 2023). Consequently, glacier mass loss has occurred at an enhanced rate, particularly around smaller ice masses in the APR and sub-Antarctic islands (Oliva et al. 2017; Engel et al. 2018; Rosa et al. 2020). This ice mass loss has resulted in the enlargement of proglacial regions, and they will continue to expand as both land- and marine-terminating glaciers continue to retreat with a warming climate (Nedbalová et al. 2013; Lee et al. 2017; Roman et al. 2019).

In this study we will map the land cover of six major proglacial regions in Antarctica: (1) South Georgia, (2) southern Livingston Island and Snow Island (hereafter referred to as Byers Peninsula), (3) Deception Island, (4) James Ross Archipelago, (5) Alexander Island, and (6) the McMurdo Dry Valleys (Figure 1). These sites are conspicuous for their lack of consistent land cover data between the sites. Though geological and geomorphological studies have produced maps at the sites (e.g., Table 1), they lack a common nomenclature. Similarly, many of these maps are several decades old or no map of their surface exists. On Alexander Island, for example, very few descriptions of the landscape or land cover are available, with limited descriptive accounts (Heywood, Fuchs, and Laws 1977) and only very limited geomorphology maps of the region available (M. C. Salvatore 2001). In contrast, some regions have been the subject of extensive mapping studies. James Ross Island, for example, has been home to several geological and geomorphological surveys, though these studies are either limited to the Ulu Peninsula (Davies et al. 2012; Mlčoch, Nývlt, and Mixa 2020; Jennings et al. 2021) or lack detail on land cover information beyond the geology (Smellie 2013). Though there have been recent, substantial, efforts in improving the understanding of vegetation extent in Antarctica (Walshaw et al. 2024), there continues to be a lack of understanding of other import land features.

**Table 1.** Resources used to interpret clusters and assign them to a land class.

Location	Resources
James Ross Island	Geomorphology map, Jennings et al. (2021) Geomorphology map, Davies et al. (2013) Geological map, British Antarctic Survey, Smellie (2013) Geological map, Czech Geological Survey, Mlčoch, Nývlt, and Mixa (2020) Vegetation map, Barták et al. (2015)
Dry Valleys	Interactive geological map, SCAR, Cox et al. (2023)
Alexander Island	Geological map, British Antarctic Survey (1981)
Deception Island	Geology and geomorphology map, British Antarctic Survey, Smellie et al. (2002) ASPA 140 (map of vegetation), Secretariat of the Antarctic Treaty (2022)
Livingston Island	Geomorphology map, Lopez-Martinez et al. (1996) Vegetation map, Ruiz-Fernández et al. (2017)
South Georgia	Geomorphology map, Clapperton (1971)

Understanding the makeup of Antarctica’s proglacial regions and how those land surface components are changing is important because they are a source of water, sediment, and solutes. The quantity and spatiotemporal pattern of sediment discharged from Antarctica have profound effects on the ecosystem of the Southern Ocean and polar lakes, which in turn can affect the rate at which carbon is sequestered from the atmosphere (Brussaard et al. 2008; Maat, Visser, and Brussaard 2019). Additionally, changes in vegetation cover can have wide-ranging impacts on wildlife. In a warming climate, the natural range of indigenous species may increase (Convey and Smith 2007). Similarly, people visiting the APR and sub-Antarctic may introduce invasive species (Galera et al. 2021; Tejedo et al. 2022). The establishment of invasive species can expand the vegetated area, displace indigenous biota, increase competition, and alter food web linkages, potentially threatening the survival of indigenous species (Molina-Montenegro et al. 2012; Hughes et al. 2020). It is therefore important to have a baseline data set that describes the land cover composition of proglacial landscapes (Carrivick et al. 2018, 2019) so that future changes may be quantified. Furthermore, understanding how proglacial landscapes have responded to recent ecological and climatic change is useful for understanding how these systems may evolve in the future (Wilkes et al. 2023).

The aims of this article are to (1) produce the first unified map of land cover across the major proglacial areas of APR, sub-Antarctic, and the Dry Valleys; (2) quantify the overall accuracy of our data and how that accuracy varies spatially; and (3) identify regions that have changed during the twenty-first century.

### Study sites

There is a dearth of literature that seeks to characterize proglacial regions, particularly in Antarctica. Some research has been conducted on individual rivers and catchments, notably on the Onyx River (Chinn and Mason 2016), James Ross Island’s Ulu Peninsula (Davies et al. 2013; Nedbalová et al. 2013; Kavan et al. 2017; Jennings et al. 2021; Kavan 2021; Sroková and Nývlt 2021), and other sub-Antarctic islands, such as the South Shetland Islands (Mink et al. 2014; Oliva et al. 2016). However, these studies have taken varying approaches to characterizing landscape compositions, and there is little in the way of a consistent land cover data set of these proglacial regions. Additionally, important global data sets fail to characterize the land cover of Antarctica (e.g., Brown et al. 2022).

#### Climate

All six study sites have polar climates but span both maritime and continental settings. The sites are positioned along a latitudinal gradient and so permit an analysis of land cover variability with climatic patterns. The most northern site, South Georgia, is characterized by its high relief and has a mean annual air temperature (MAAT) of 3°C, as well as receiving over 2,000 mm of precipitation per year (Bannister and King 2015; Strother et al. 2015). Over half of South Georgia is glacierized (Bannister and King 2015). The South Shetland Islands are characterized by a polar maritime climate, with air temperatures regularly exceeding 0°C in summer. The humid environment, due to its maritime location, ice-free seas, and regular cyclonic activity, results in liquid precipitation falling regularly in the summer months (Bañón et al. 2013). The James Ross Archipelago, to the northeast of the Antarctic Peninsula, has an MAAT of −7°C and has a semi-arid polar continental climate (Kaplan Pastíriková et al. 2023). The two more southerly sites, Alexander Island and the McMurdo Dry Valleys, have continental climates (Harangozo, Colwell, and King 1997). Alexander Island, specifically Fossil Bluff, has an MAAT of −9°C and receives approximately 200 mm of precipitation each year (Harangozo, Colwell, and King 1997; Davies et al. 2017). The McMurdo Dry Valleys are distinctly colder and drier than the other sites; they are hyper-arid due to katabatic winds and have an MAAT of −17°C to −20°C (Doran, Wharton, and Lyons 1994; Marchant and Head 2007).

## Methodology

### Site selection

Our site selection was informed by the British Antarctic Survey’s rock outcrop data sets (Burton-Johnson et al. 2016; Gerrish, Fretwell, and Cooper 2020), allowing us to focus primarily on the nonglacierized landscape. Nunataks in the interior of the ice sheets were excluded because they were too small to classify at 30-m resolution, and we could assume their classification to be bedrock. Because they are disconnected from the coastline, they can also be assumed largely unimportant as sediment sources to the Southern Ocean. Fossil Bluff and other coastal regions on Alexander Island and Palmer Land were included and are interesting for their proximity to George VI Sound. These regions may become important sediment sources in the near future, because exceptional melting in this region appears to have increased the likelihood of the George VI ice shelf collapsing (Banwell et al. 2021). We further narrowed the site choices to consider only those regions with cloud-free Landsat 8 Operational Land Imager (OLI) images.

### Land cover classifications

In the last decade, satellite data from the Landsat and Sentinel programs have become open source and increasingly easy to access. In tandem with improved computational power, such as that provided by cloud-based platforms like Google Earth Engine (GEE), it is now possible to produce land cover maps at a medium spatial resolution (10–30 m) using openly available data. The Landsat 8 satellite also has the benefit of being part of a continuation program, making interdecadal comparison possible.

#### Image selection and pre-processing

We classified Landsat 8 OLI (top-of-atmosphere) images acquired between 2016 and 2020 (see Supplementary Material Section 1.6 for details) in GEE and ESRI ArcGIS Pro 2.6.0, primarily using *K*-means clustering (using GEE’s default settings, including ten randomized seeds). Though we have chosen to use GEE and ArcGIS Pro 2.6.0 for this research, it would be functionally possible to repeat our methodology using other software. We chose Landsat imagery, rather than higher-resolution images (such as Sentinel-2), because of its extensive archive dating back to 1972. Suitable images had low cloud cover (less than 20 percent over land) and limited snow cover. Images were cloud masked (using Landsat’s quality assessment band) and, where more than one image was available, we mosaicked them, taking the least cloudy/snowy scene as the uppermost image, thus minimizing the snow and cloud cover across the unified scene.

To ensure consistency with older Landsat images, we only selected six bands representing the visible and infrared wavelengths (red, green, blue, near-infrared, shortwave infrared 1, and shortwave infrared 2, ranging from 0.45 to 2.29 μm) from the images for classification. We added three further bands to the image in the form of the Normalized Difference Snow Index (NDSI; Equation (1)), the Normalized Difference Vegetation Index (NDVI; Equation (2)), and the Normalized Difference Water Index (NDWI; Equation (3)). These aided the classifier in the identification of key land cover classes (ice, vegetation, and water, respectively).

(1) $$\begin{array}{c} \mathrm{NDSI}=\;\frac{\mathrm{green}\;-\;\mathrm{swir}1}{\mathrm{green}\;+\;\mathrm{swir}1} \end{array}$$

(2) $$\begin{array}{c} \mathrm{NDVI}=\;\frac{\mathrm{nir}\;-\;\mathrm{red}}{\mathrm{nir}\;+\;\mathrm{red}} \end{array}$$

(3) $$\begin{array}{c} \mathrm{NDWI}=\;\frac{\mathrm{green}\;-\;\mathrm{nir}}{\mathrm{green}\;+\;\mathrm{nir}} \end{array}$$

where

- green = band 3 of Landsat 8 OLI, wavelength (λ) = 0.53 to 0.59 μm
- swir1 = shortwave infrared 1, band 6, λ = 1.57 to 1.65 μm
- red = band 4, λ = 0.64 to 0.67 μm
- nir = near-infrared, band 5, λ = 0.85 to 0.88 μm

We clipped the images to a 1-km buffer around their coastline (Gerrish, Fretwell, and Cooper 2021) and topographically corrected them to adjust for the effect of relief on the illumination of images using the Sun Canopy Sensor + C method (Soenen, Peddle, and Coburn 2005) with the Reference Elevation Model of Antarctica mosaic digital surface model (Howat et al. 2019) at 30-m resolution (equivalent to the resolution of Landsat 8 OLI multispectral bands). South Georgia, which is not covered by Reference Elevation Model of Antarctica, was corrected using the Shuttle Radar Topography Mission Digital Elevation Model, also at 30-m resolution (Farr et al. 2007). Subsequently, we conducted a principal component analysis of the images, and the first three components, containing 99.6 percent (±0.3 percent) of the data, were selected for classification (Frohn et al. 2009; Chasmer et al. 2020).

#### Classification

**Figure 2.**
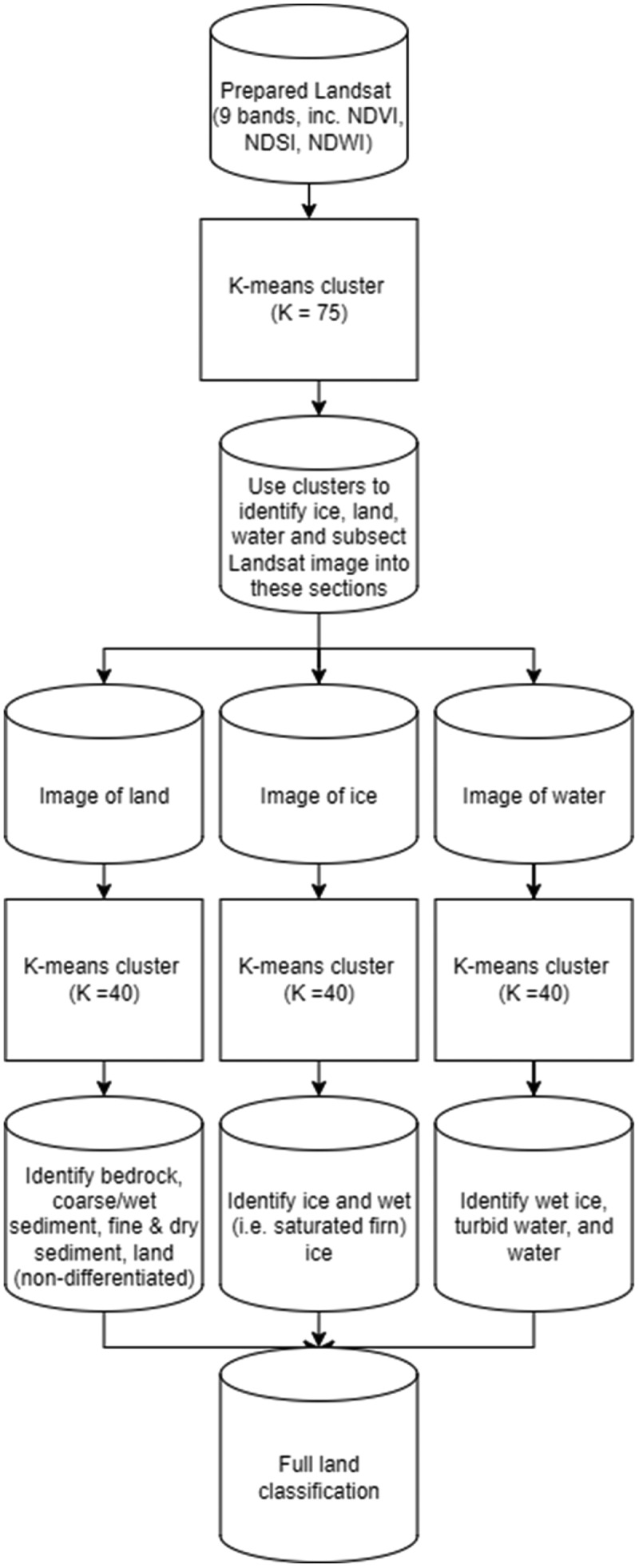
Our approach to classifying land cover.

We used a hierarchical *K*-means clustering approach to classify Landsat 8 OLI images (Figure 2). *K*-means is widely used in land classification studies (Phiri and Morgenroth 2017; Grimes et al. 2024) and is preferential to over other unsupervised approaches (e.g., ISODATA) because it can be used to identify a user-defined number of classes. *K*-means works by segmenting an image into distinct clusters, which the user then interprets to classify these clusters using existing knowledge of the field or previously published maps often based on field research (e.g., Table 1). A first-order land classification (clustered with *K* = 75, see Supplementary Material Section 1.1) of “land,” “snow and ice” (hereafter referred to simply as “ice”), and “water” informed the subdivision of each of these classes in a second, more detailed, analysis of the dominant land cover classes (further details in Supplementary Material Section 1.2.). A two-stage approach was used to limit misclassification by ensuring that water, ice, and bare land were in distinct classes. The code used to produce this classification is also publicly available (see section 2.4.5).

We used this first-order land classification to subset each image accordingly and then to cluster these resulting images into fory discrete groups (*K* = 40). Specific *K* values were determined through expert judgment and represent values that minimized the chance of misclassification (see further details in Supplementary Material 1.1). Using the limited catalog of published maps and literature available for these areas (see Table 1), we visually inspected these clusters to manually assign each of them a final land classification. Our first-order land class was subset into five classes: “bedrock,” “coarse/wet sediment,” “fine & dry sediment,” “vegetation,” and “land (nondifferentiated).” The water class was subset into “water” and “turbid water,” and the ice class was subset into “ice” and “wet ice.” In cases where clouds partially obscured land, we assigned pixels to the more general class of “land (nondifferentiated).” Therefore, we produced ten land classes that describe eight distinct surface types (plus no data and land undifferentiated; see Supplementary Material for more details) that could be identified from a combination of field observations and a review of available maps of Antarctica (Table 1) and finding commonalities between them (further details in Supplementary Material Section 1.3.).

During the classification process, we created two different sedimentary classes because we found that pixels containing wet sediments (such as rivers) or blocky superficial sediments, such as scree, clustered distinctly from those pixels that contain sediments smaller than cobbles in size and fissile sedimentary rocks. This approximate grain size threshold was derived from information on geomorphological maps for the region (Jennings et al. 2021) and observations made on James Ross Island during the 2022 field season. We emphasize that the first of these two classes describe pixels that contain sediment that may be coarse, wet, or both. The second of these classes describes surfaces with fine sediments with minimal water content.

### Accuracy assessment

Having used the limited preexisting maps and field survey data to inform our interpretation of the *K*-means clusters, we had to depend on finer-resolution imagery as the primary independent validation source, with interpretation of images aided by the use of previously published maps. Although we could not find alternative land cover data, we still used the methods of best practice described by Olofsson et al. (2013, 2014) to that ensure our accuracy assessment was robust (see Supplementary Material Section 1.5). Therefore, we generated 3,000 random points, stratified by the area of each land class, and visually compared them to 10-m resolution Sentinel-2 MultiSpectral Instrument images. Sentinel-2 MultiSpectral Instrument images were used as an independent data source for validation because they are finer resolution than Landsat images, thus giving a better indication of the “true” land cover. Given the dominance of the ice class in our classification, this meant that most of the stratified sample points landed on ice. We conducted a second level of accuracy assessment with 1,000 points on just the proglacial classes to ensure that their accuracy was adequately calculated.

The classes of turbid water and wet ice were particularly problematic because they typically comprised episodic sediment plumes and snow/ice melt. Therefore, we combined these classes with water and ice respectively for the purposes of accuracy assessment. We produced a 10-km-resolution grid to display the spatial variability in the accuracy of this classification (as a proxy for confidence), with each cell color-coded according to the percentage of accurate assessment points within it. Full accuracy assessment matrices are available in the supplementary material (Section 1.5).

We also compared the spectra for each land type to ensure that each land type could reasonably be differentiated from the others.

### Change detection

**Figure 3.**
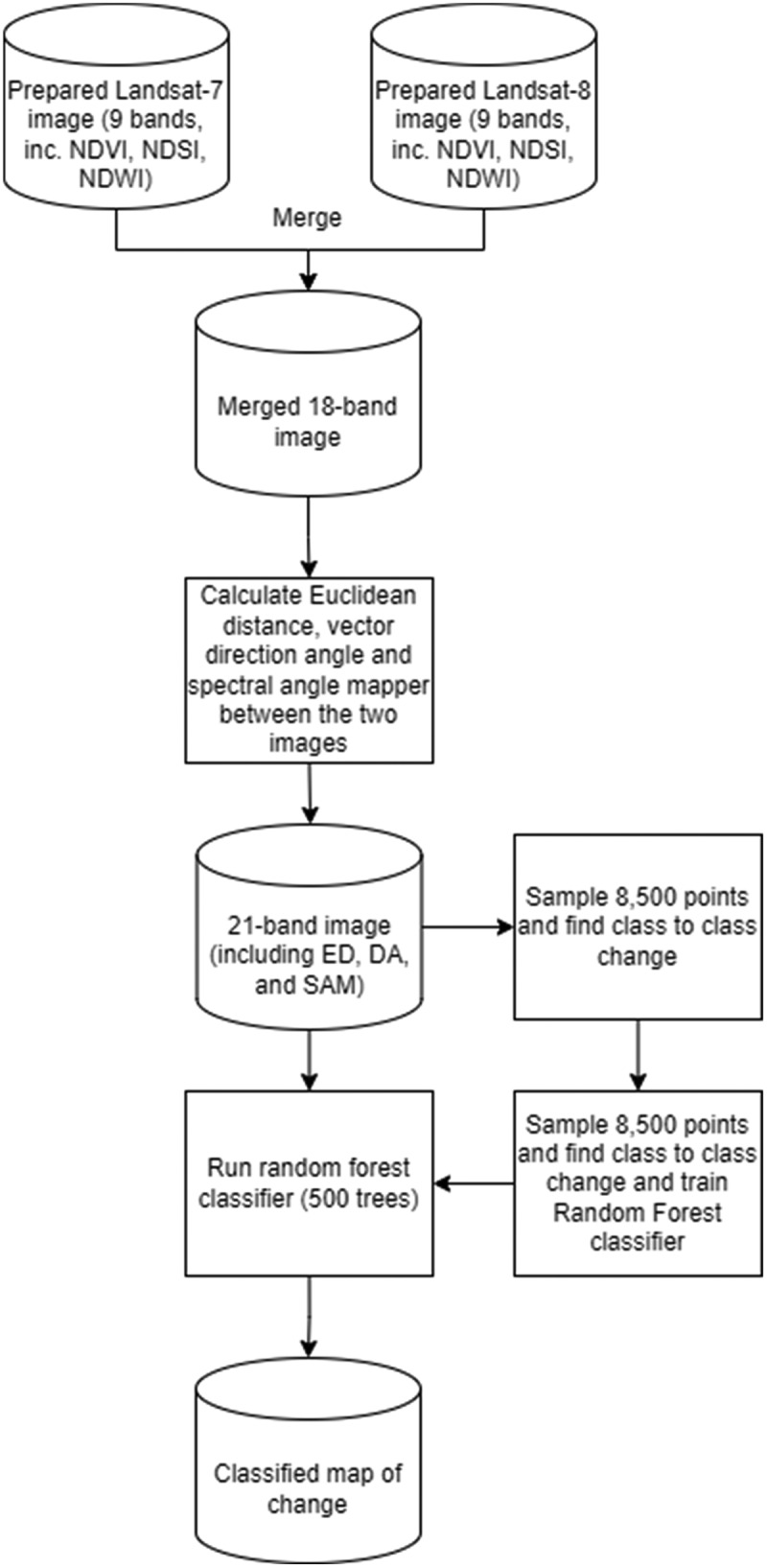
The change detection (CVA) approach used in this study.

We repeated the search described in section 2.2.2 for Landsat 7 Enhanced Thematic Mapper Plus (ETM+) images acquired for each of our sites between 2000 and 2003 and conducted change detection (Figure 3). This search resulted in a pair of image mosaics (hereafter referred to as image pairs) for five sites, comprising a mosaic from the early 2000s (Landsat 7) and a mosaic from close to 2020 (Landsat 8). It was not possible to find a suitable image for Deception Island, so we could not conduct change detection for this site; this meant that change detection was conducted over only five of the six sites for which a land cover map was produced. We manually inspected the image pairs for each site to ensure that they were co-registered using geographic information systems. We aimed to ensure that both mosaics comprised images collected from the same time of year to ensure that they represented the same part of the growth and hydrological season and avoided images with high snow cover, where possible. In some cases, poor image availability meant that some image pairs could not be collected from the same time of year (though the temporal difference was minimized). We ensured that key features such as flowing rivers and unfrozen lakes were, as much as possible, present in both mosaics. Then we conducted a change vector analysis (CVA) to identify regions of change in each of our sites using the approach described by (Xu et al. 2018). Further details of the CVA approach used can be found in the supplementary material (Section 1.4).

#### Accuracy assessment

To validate the accuracy of our change maps, we reproduced the change detection analysis on Byers Peninsula with a 70:30 split of the training points between the classifier and validation. This approach is regularly used to assess the accuracy of land cover and change products, in the absence of independent data (Xu et al. 2018), and this ratio between training and validation has been shown to be most reliable (Adelabu, Mutanga, and Adam 2015). By splitting the data 70:30 between training and validation, the 30 percent of pixels used for validation are “independent” of those used by the classifier. To ensure that this split was unbiased, we randomly sorted the training points.

## Results and interpretations

### Land cover classifications

#### The land classes

**Figure 4.**
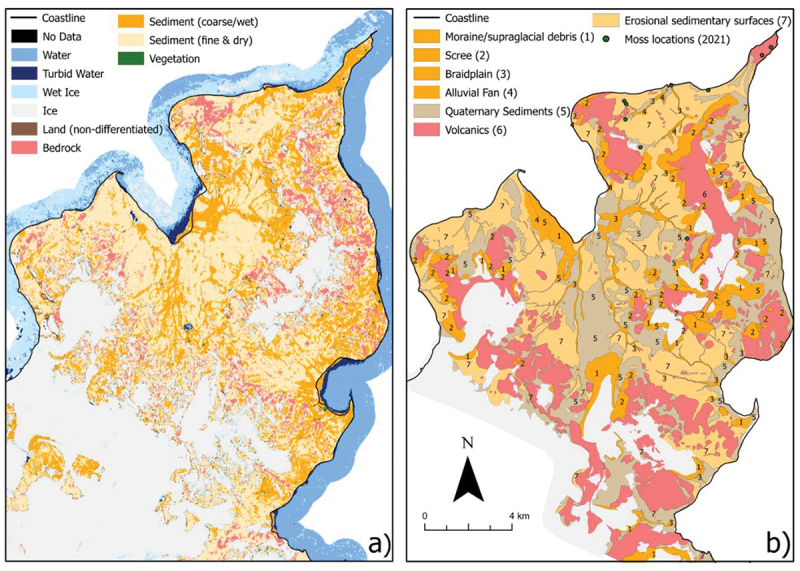
A comparison between (a) the land classification produced in this study and (b) a geomorphology map, adapted from Jennings et al. (2021). Jennings et al. (2021) produced these data through a series of extensive field surveys on the Ulu Peninsula. Vegetation locations as collected in the field by Jan Kavan (of the Czech Antarctic Research Programme) in 2021 are also displayed. Note the similarities in the ice class, locations of river systems, and scree slopes. The colors in (b) have been adapted to allow a more direct comparison with the map produced in this study (a).

The largest land class at our sites is ice; the large ice sheets and glaciers at all sites have been mapped, though this class also includes limited snow cover. Though mapping ice masses is not the primary goal of this study, the high accuracy (see section 3.3.1) of the ice class makes this data set a useful resource to assess changes in the small land-terminating glaciers within our study sites (Figure 4).

**Figure 5.**
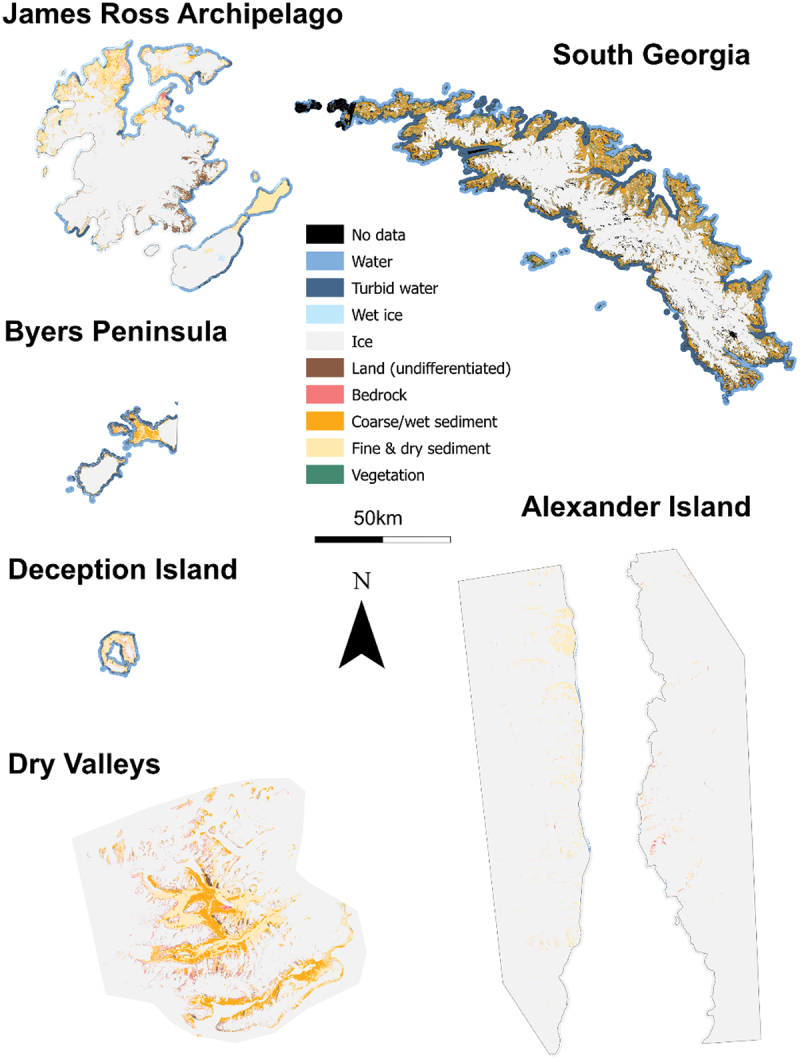
Land cover maps of the six sites, including ten classes that describe eight distinct surfaces. Ice class may include limited areas of seasonal snow cover. Higher resolution maps can be found in the supplementary material (Section 2).

**Figure 6.**
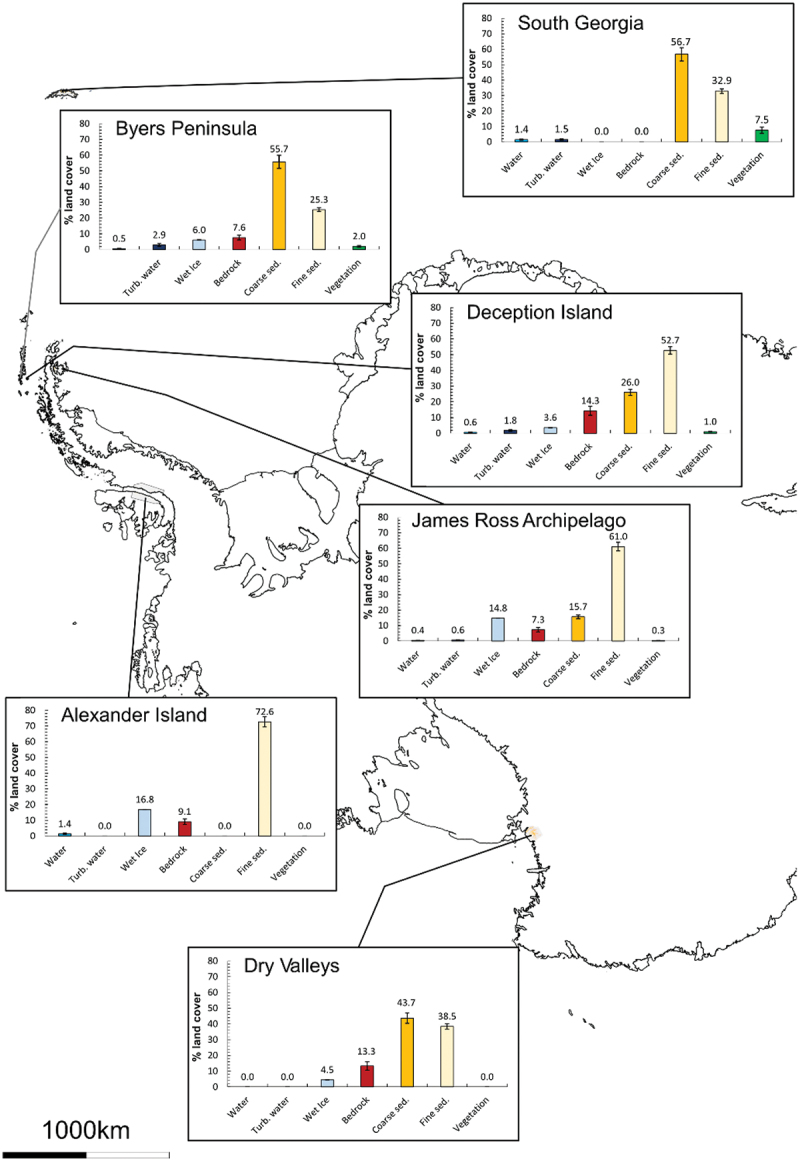
Percentage land cover values (excluding ice, no data, and land [undifferentiated]) for each site, overlaying the coastline of Antarctica (coastline sourced from BAS). Error bars indicate the 95 percent confidence intervals.

Of the sedimentary classes, coarse and wet sediment is the predominant land class at four of the six sites, particularly on South Georgia and Byers Peninsula, where it represents the majority (57 and 56 percent, respectively) of the proglacial land cover (Figures 5 and 6). This land class includes the major surface drainage networks of Antarctica (Figure 4); for example, it accurately depicts the major rivers of the Bohemian Stream and Abernethy River on James Ross Island and the Onyx River in the McMurdo Dry Valleys (cf. Chinn and Mason 2016; Kavan et al. 2017; Jennings et al. 2021). The coverage of fine and dry sediment class varies inversely to that of the coarse/wet sediment. For example, on South Georgia, the 57 percent coverage of coarse sediment is in comparison to a 33 percent coverage of fine and dry sediment. On Deception Island, where fine and dry sediments are the dominant land class (53 percent), there is only 26 percent coverage of coarse/wet sediment (Figure 6). At all of the sites, between 70 and 80 percent of the proglacial surface is covered by sediment. The bedrock class, which primarily describes igneous and metamorphic rock surfaces, is most abundant on Deception Island, comprising 14 percent of its proglacial areas (Figure 6). It is of similar abundance in the Dry Valleys (13 percent), with between 7 and 9 percent of Alexander Island, James Ross Archipelago, and Byers Peninsula comprising bedrock. The absence of the bedrock class on South Georgia is accounted for by its lack of igneous outcrops, as well as well-developed sedimentary systems and extensive vegetation cover (Clapperton 1971).

**Figure 7.**
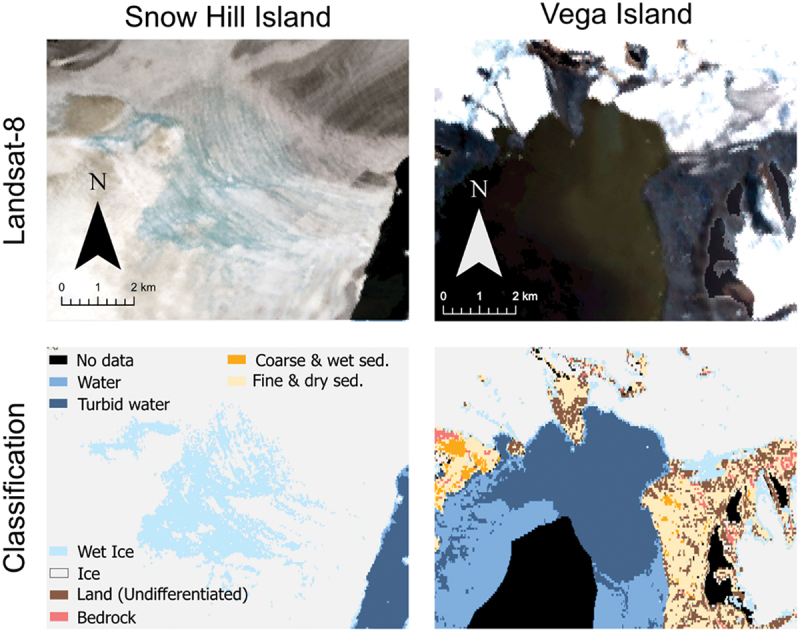
How the wet ice and turbid water classes compare to the images they are derived from, with a large area of saturated firn on Snow Hill Island (64°28′ S, 57°4′ W) and a sediment plume off the coast of Vega Island (63°52′ S, 57°16′ W).

The classes relating to water (water, turbid water, wet ice) are of varying quantities across all of the sites and may represent transient features (e.g., seasonal meltwater/sediment plumes). The wet ice class proved to be a little ambiguous to interpret from clusters and represents saturated firn and “slush” ice (i.e., partially melted ice or partially frozen water). Wet ice is most abundant on Alexander Island, with 17 percent coverage (Figure 6), and highlights the record-high surface melt observed around the King George VI Ice Shelf in late 2019 (Banwell et al. 2021). This large amount of wet ice is comparable to the James Ross Archipelago (15 percent), where a large proportion of wet ice is accounted for by a melt event that resulted in a large area of saturated firn on Snow Hill Island (Figure 7). This transient nature of wet ice is also seen with the turbid water class, which can pick out sediment plumes (Figure 7).

Our land classification has also identified regions of vegetation. This includes extensive areas of vegetation on South Georgia, which we have calculated to cover 8 percent of its proglacial surface and are clearly identifiable in satellite images (Figure 6). We have also identified several sites of vegetation on the South Shetland Islands; especially those on Deception Island (total 1 percent surface coverage; Figure 6) within ASPA 140 (subsite B) on Deception Island (Secretariat of the Antarctic Treaty 2022). In some cases, we have even been able to identify very small areas of vegetation such as those located on James Ross Island, which were verified in the field (Figure 4).

#### Spatial variations

We observe a spatial variation in land cover between the sites (Figures 5 and 6). There is typically more coarse/wet sediment at sites further away from the pole; this is offset by a general decrease in fine and dry sediments. However, the Dry Valleys are an exception to this, with 44 percent of the land covered by coarse or wet sediments. The second most southern site, Alexander Island, has 0 percent of its proglacial surface covered by coarse/wet sediment, compared with 57 percent on South Georgia.

Unlike other land classes, the proportion of the (inland) water and wet ice classes appears to be more evenly spread across the sites. There is a slight apparent latitudinal pattern in these data, with more water at the sites further to the north and variability between the east and west (i.e., when comparing the South Shetland Islands with James Ross Archipelago; Figure 6). South Georgia and Byers Peninsula have the largest amount of liquid water present (when joining the water and turbid water classes together), around 3 percent. James Ross Archipelago has significantly less (1 percent), and 1 percent of Alexander Island’s surfaces is classified as water, owing to a large amount of supraglacial water at the time of image acquisition. We classified some of this melt as water, rather than wet ice, because it was unambiguously liquid when we inspected and interpreted the clusters. Much of these intersite differences in liquid water likely represent differences in climatic setting; those sites with the greatest proportion of the water class are in milder, maritime climates, with higher temperatures and more precipitation falling as rain. The bedrock class does not show a clear latitudinal pattern and is most abundant in Deception Island (14 percent) and the McMurdo Dry Valleys (13 percent).

We noted a latitudinal pattern in the presence of vegetation, with the largest proportions of vegetation coverage observed on South Georgia and the South Shetland Islands and no coverage on Alexander Island or the McMurdo Dry Valleys. This is consistent with observations made in Arctic regions, where regions closer to the poles have significantly less vegetation coverage (Walker et al. 2018). Although no vegetation was detected on Alexander Island or in the McMurdo Dry Valleys, small areas of vegetation have previously been described (Heywood, Fuchs, and Laws 1977; Pannewitz et al. 2003), though they are typically below the resolution of our classification. The most northern site of South Georgia had significantly more vegetation than any other site (7 percent of the proglacial regions are covered by vegetation; Figure 6), whereas the McMurdo Dry Valleys and Alexander Island have no detectable vegetation coverage. James Ross Island has very little vegetation cover (<1 percent), whereas the South Shetland Islands show 2 percent coverage on Byers Peninsula and 1 percent on Deception Island.

#### Potential drivers of variability

The spatial pattern in sedimentary classes is consistent with the expectation that greater runoff should occur in polar regions with higher temperatures (Syvitski 2002). Increased runoff would result in a greater proportion of the surface being covered by the coarse/wet sediment class. However, the Dry Valleys are an exception to this, with 44 percent of the land covered by coarse or wet sediments (Figure 6). This is likely due to the high relief of the region, allowing for greater mass movement and scree formation (Kirkby and Statham 1975; Doran et al. 2002), and consistent solar radiation during the austral summer facilitating glacier melt and, in combination with subglacial drainage, the formation of large rivers such as the Onyx River (Gooseff et al. 2011; Conovitz et al. 1998; Badgeley et al. 2017). We did not identify any coarse sediment on Alexander Island. The reasoning for this is twofold: (1) an apparent lack of major drainage networks and (2) the scree slopes in this region appear to be small and thin. When viewed from Sentinel-2 images, we could identify only small-size scree slopes and very few streams, consistent with observations made by Heywood, Fuchs, and Laws (1977), who noted that many scree slopes were composed of fine sediments.

The spatial patterns in the wet ice, water, and turbid water classes show more water at the sites further to the north and variability between the east and west, likely due to climatic conditions favoring liquid water on the South Shetland Islands and South Georgia. The disproportionately large amount of water and wet ice on Alexander Island and the James Ross Archipelago relates to the high melt in these areas at the time of image acquisition (Banwell et al. 2021). The bedrock class is most abundant on Deception Island and McMurdo Dry Valleys, owing to ongoing volcanism on Deception Island (Smellie et al. 2002; Rosado et al. 2019) and extensive volcanic history of the McMurdo Dry Valleys (Petford and Mirhadizadeh 2017; Smellie and Martin 2021). This class is also associated with volcanic rocks on James Ross Island (Mlčoch, Nývlt, and Mixa 2020; Jennings et al. 2021) and Byers Peninsula (Gao et al. 2018) and metamorphic rock outcrops on Alexander Island (British Antarctic Survey 1981).

Though latitude accounts for some of the variation in vegetation coverage, it is not the only factor. The sparse vegetation coverage on James Ross Island, despite its relatively low latitude, is consistent with field observations and is logical given its semi-arid climate and high wind speeds (Martin and Peel 1978; Davies et al. 2013; Barták et al. 2015; Nývlt et al. 2016; Hrbáček and Uxa 2020; Kňažková, Nývlt, and Hrbáček 2021; Váczi and Barták 2022). The relatively high vegetation coverage of Byers Peninsula and South Georgia is also logical given the milder maritime climates of the South Shetland Islands and South Georgia, compared to the drier continental climate of Alexander Island and the McMurdo Dry Valleys. Deception Island has less vegetation than the neighboring Byers Peninsula, perhaps due to the impact of ongoing volcanic activity on the island and relatively recent eruptions resulting in unfavorable conditions (Collins 1969; Smith 1988, 2005).

### The changing landscape

**Figure 8.**
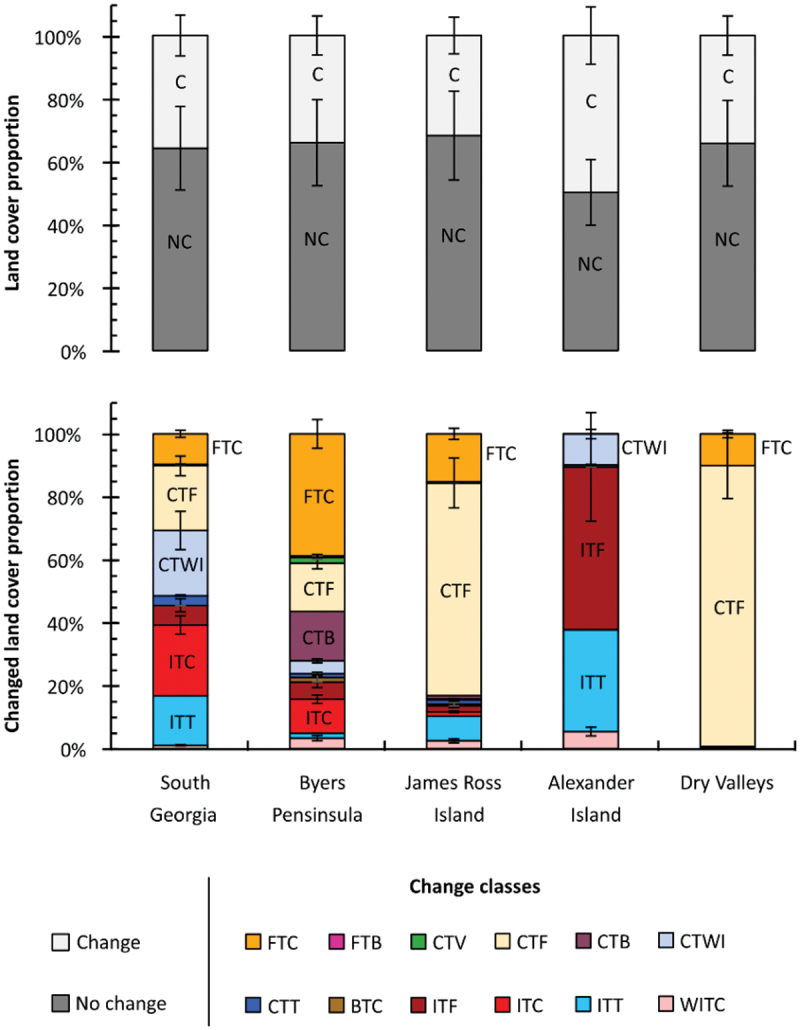
The proportion of the proglacial landscape that has changed at each site analyzed and the makeup of those changed regions.

Out of the five sites we investigated for change, four had similar landscape stability with between 64.2 and 68.2 percent of the land cover remaining unchanged during our study period (Figure 8). Alexander Island, however, varies from this trend with a no change proportion of just 50.2 percent. This is primarily due to the exceptional melt of snow and ice in the region at the time of the second image (2019), which led to more sediment being exposed (ITF) and some lakes and supraglacial lakes (ITT; see Table 2) forming in their place. Eighty-four percent of the change on Alexander Island is due to loss of the ice class, associated with snow and ice melt. This dramatic change in land cover coincides with sustained positive-degree temperatures that occurred in 2019 for the contemporary image and also led to exceptional melt on the George VI ice shelf (Banwell et al. 2021).

**Table 2.** Class-to-class changes and their abbreviations.

Class-to-class change	Abbreviation
Wet ice to coarse/wet sediment	WITC
Ice to fine and dry sediment	ITF
Ice to coarse/wet sediment	ITC
Ice to turbid water	ITT
Coarse/wet sediment to turbid water	CTT
Coarse/wet sediment to wet ice	CTWI
Fine and dry sediment to bedrock	FTB
Coarse/wet sediment to bedrock	CTB
Coarse/wet sediment to fine and dry sediment	CTF
Coarse/wet sediment to vegetation	CTV
Bedrock to coarse/wet sediment	BTC
Fine and dry sediment to coarse/wet sediment	FTC

Alexander Island is also the exception to a general pattern we observe in the loss of ice across Antarctica. In general, there is a latitudinal pattern in the loss of ice across our sites. If we consider the ITT, ITC, and ITF classes, 45 percent of South Georgia’s land cover change is associated with ice loss. In contrast, this value was less than 1 percent for the Dry Valleys, a two orders of magnitude difference. This pattern of ice loss occurs in tandem with a southward increase in the proportion of land cover change associated with sedimentary changes (FTC, CTB, or CTF). Some of these differences in sedimentary class may also be accounted for by the stabilizing and moisture-retaining properties of vegetation coverage (Aalto, le Roux, and Luoto 2013; Klaar et al. 2015), which is higher at the more northerly sites (Figure 6). If we specifically consider the FTC class, we see that it is most abundant on Byers Peninsula. This is likely a product of episodic changes in the flow of streams, which would be expected in the South Shetland Islands given their high rates of precipitation (Bañón et al. 2013). Of the three sites where vegetation was identified in the land cover product, the greatest change was seen on the Byers Peninsula; with 2 percent of its total change accounted for by the CTV class, exceptional vegetation growth in the South Shetland Islands is consistent with previous findings (Torres-Mellado, Jaña, and Casanova-Katny 2011).

### Data accuracy

#### Overall accuracy of land cover product

The overall accuracy of our land cover classification is 95.9 percent. However, this overall value should be taken with caution, because a large proportion of our areas of analysis are covered by ice. This high accuracy represents the fact that our approach is very effective at differentiating ice from land and water. The accuracy of each land class individually provides a more informative assessment of this approach. We find that each proglacial land class has a relatively large standard error, owing to the small number of pixels that we checked (Table 3).

**Table 3.** Accuracy assessment of all land classes.

Class	Error-adjusted area (km^2^)	95 Percent confidence (km^2^)	Percentage area	Percentage error	n
Water	99.1	45.5	0.2	45.9	9
Ice	44,001.5	219.6	92.2	0.5	2,595
Bedrock	231.3	174.2	0.5	75.3	27
Fine and dry sediment	2,131.5	195.9	4.5	9.2	134
Coarse/wet sediment	1,156.6	174.2	2.4	15.1	114
Vegetation	115.7	56.4	0.2	48.8	10

*n* < 3,000 because several points landed on cloud-covered parts of the reference images. Percentage error refers to the size of the 95 percent confidence bounds, relative to the error-adjusted area.

The overall accuracy of the proglacial component of the classification is 77.0 percent, with the greatest percentage uncertainty in the smaller-sized land classes (water and vegetation). Though this overall accuracy is slightly lower than some products (e.g., Malinowski et al. 2020; Pazúr et al. 2022), it should be noted that we achieved this without the availability of extensive training data, making it more comparable with the more moderate accuracies achieved by Chen et al. (2015), for example. The sediment classes typically perform well, with relatively small percentage errors (Table 4). The confusion matrices can be found in the supplementary material (Section 1.5).

**Table 4.** Accuracy assessment of proglacial classes.

Class	Error-adjusted area (km^2^)	95 Percent confidence (km^2^)	Percentage area	Percentage error	n
Water	85.7	26.4	2.0	30.9	15
Bedrock	285.5	56.7	6.6	19.9	45
Fine and dry sediment	2,375.5	106.9	54.7	4.5	371
Coarse/wet sediment	1,444.7	108.8	33.3	7.5	257
Vegetation	148.5	40.1	3.4	27.0	34

*n* < 1,000 because several points landed on cloud-covered parts of the reference images. Percentage error refers to the size of the 95 percent confidence bounds, relative to the error-adjusted area.

Because we were unable to assess the accuracy of the turbid and wet ice classes, we have provided an example of a classification of each land class, to allow for a qualitative assessment of its accuracy (Figure 7).

When comparing the spectra, we found that our identified classes had distinct spectral signatures that were consistent between locations (Supplementary Section 1.7). Some subtle differences, mostly within the red and near-infrared bands, existed in the sediment and bedrock classes and most likely represent differences in regional geology (M. R. Salvatore et al. 2014). The pattern for vegetation is also notable. Vegetation is typically characterized by peaks in the near-infrared wavelengths; however, we do not observe this in our spectra, likely because the vegetation of Antarctica is dominated by cryptogamic species (e.g., moss), which do not reflect strongly in this band (Váczi et al. 2020). The spectra for South Georgia do show a peak in the near-infrared band, consistent with the presence of vascular (leafy) vegetation (Tichit et al. 2024).

We find that our sedimentary classes are similar in spectral pattern (likely due to similarities in geology) but that the coarse/wet class present with lower reflectance values at each site (Supplementary Section 1.7). We interpret this to be due to either its higher water content or its higher grain size (Clark 1999; M. R. Salvatore et al. 2023), which would explain the challenges we found in differentiating between coarse and wet sediments. We note that this distinction is not as clear with the classes on Deception Island. Though we have assigned *K*-means clusters to different classed based on the previously mapped presence of scree and streams, additional caution should be used for interpretations made at this site. The water (water and turbid water) classes are also distinct from each other (Supplementary Section 1.7), primarily on the basis reflectance values, consistent with previous studies showing that turbid water has higher reflectance values (Cui et al. 2022). We observed distinctly higher reflectance values for water at Alexander Island, probably because the water at this site is mostly ponded on top of glaciers/ice.

#### Spatial confidence in land cover product

**Figure 9.**
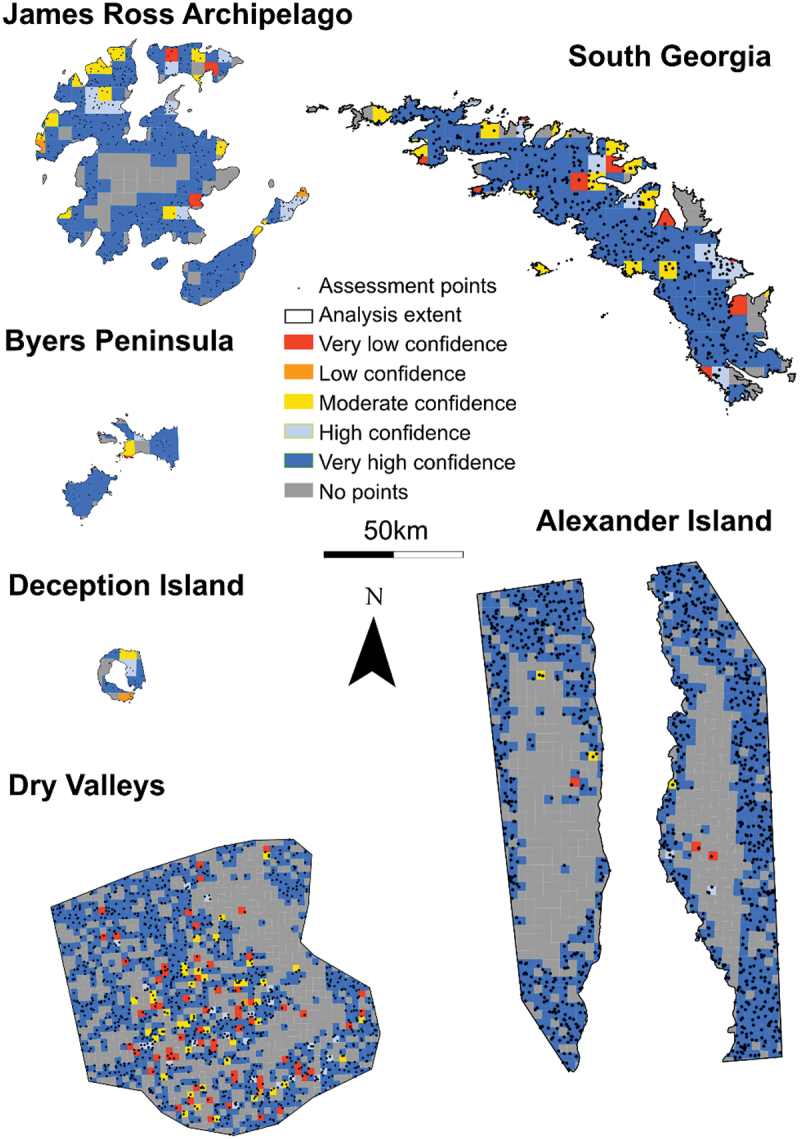
Maps of each site indicating the spatial variability in confidence. Very low confidence = <20 percent of points were accurate; low confidence = 21 to 40 percent; medium confidence = 41 to 60 percent; high confidence = 61 percent to 80 percent; very high confidence = >80 percent.

We produced a map to represent the confidence of our data set (Figure 9), which is notable for its spatial homogeneity; no individual site appears to be more or less accurate than any other. The McMurdo Dry Valleys have the most “very low confidence” cells, but this is a function of it being the second largest site analyzed, with the largest coverage of proglacial land. Because proglacial classes are less accurate than ice (Tables 3 and 4), it is to be expected that the greatest amount of very low confidence cells would be present here. We also observed that many of these very low confidence cells contain only one or two assessment points. This means that just one inaccurate point may result in the cell being classified as very low confidence, when in fact further analysis may reveal that it performs better than represented here.

We also note that the highest accuracy—that is, the regions with the highest density of “very high confidence” cells—is within the ice sheets at each site, which is consistent with the accuracy assessment (Table 3). This is particularly clear on South Georgia and Alexander Island. The regions with “no points” are primarily over the large ice sheets, particularly to the center of James Ross Island, Alexander Island, and the Dry Valleys. Because of the large coverage of ice, many cells were not checked during the accuracy assessment because the random point algorithm does not regularly space points. However, in reality, we are highly confident of cells within the center of ice sheets: they are clearly ice when inspected, and the 92.4 percent accuracy of the ice class (Table 3) suggests that they are very likely to be accurate.

### Overall accuracy of change detection

We found that our change detection approach had a total validation accuracy of 80.1 percent. The accuracy varies by class (Table 5), with the most accurate classes being ITT and FTB, albeit from a low sample size. The least accurate class is the coarse/wet sediment to wet ice (CTT) class. However, as stated in section 2.4.1, it is also important to consider the geomorphological processes that the change classes represent. For example, if we merge together classes that represents the same process as CTT (i.e., formation of a lake/formation of a wet area), the error reduces from 60.0 to 5.9 percent.

**Table 5.** Accuracy assessment of land cover change.

Change class	Geomorphological process	Percentage error	Geomorphological process percentage error	n
No change	No change	20.7	20.7	1,563
Wet ice to coarse/wet sediment	Ice melt (land)	25.0	2.8	8
Ice to turbid water	Ice melt (water)	0.0	0.0	13
Ice to coarse/wet sediment	Ice melt (land)	12.7	2.8	79
Ice to fine & dry sediment	Ice melt (land)	33.3	2.8	21
Bedrock to coarse/wet sediment	Sediment deposition	6.7	6.7	15
Coarse/wet sediment to turbid water	Lake formation^a^	60.0	60.0/5.9	10
Coarse/wet sediment to wet ice	Slush–ice formation/lake formation^a^	29.2	29.2/5.9	24
Coarse/wet sediment to bedrock	Erosion	32.3	21.6	127
Coarse/wet sediment to fine & dry sediment	Drying	15.3	15.3	98
Coarse/wet sediment to vegetation	Vegetation formation	30.8	30.8	13
Fine & dry sediment to bedrock	Erosion	0.0	21.6	1
Fine & dry sediment to coarse/wet sediment	Wetting	11.7	11.7	290

Percentage error denotes the proportion of pixels misclassified within that land class. Geomorphological process error denotes the error of the geomorphological process represented by one or more change classes.

^a^There are two possible ways in which classes can be represented as a geomorphological process: either as lake formation and slush-ice formation, or both could be represented as one lake formation class. This affects the resultant geomorphological process error; therefore, two geomorphological process errors are displayed.

**Figure 10.**
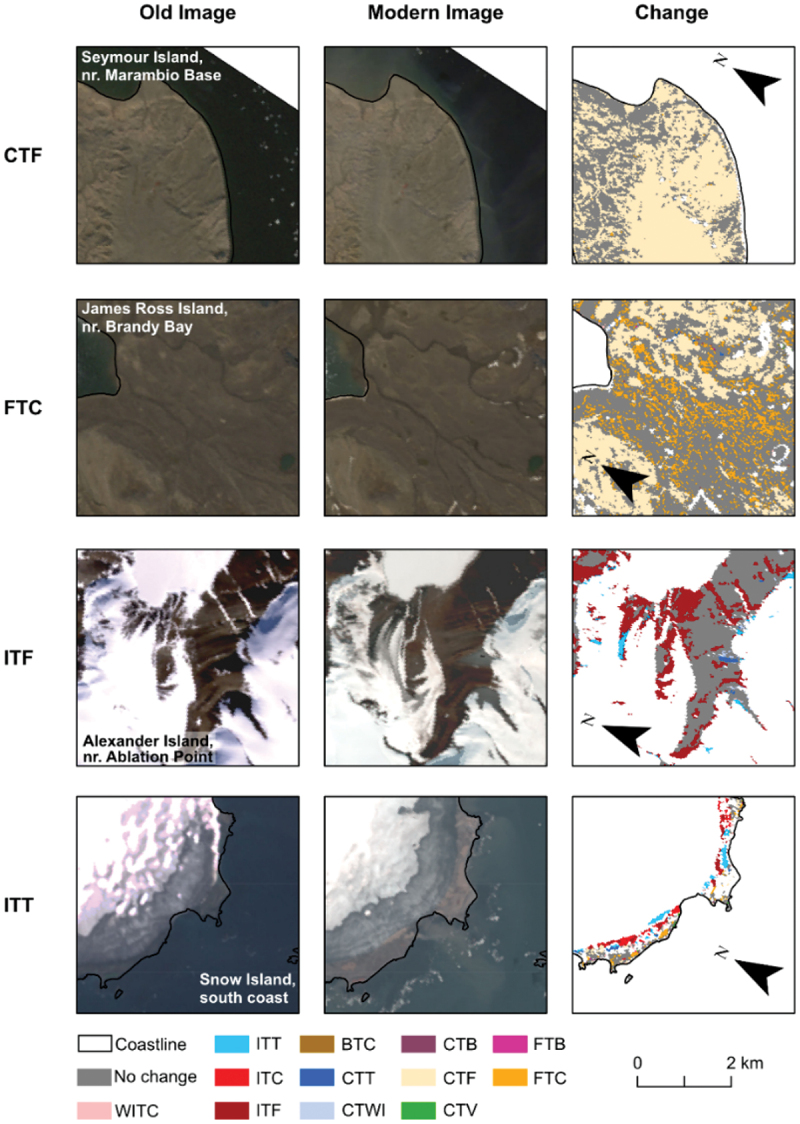
Examples of the four most frequently observed change classes: 86 percent of the change identified in out data can be described by these four classes. The CTF example shows less active river channels in the modern image associated with drier sediments on Seymour Island. FTC shows the opposite, with more active river channels associated with wetter sediments on James Ross Island. The ITF example shows a reduction in the extent of glaciers and snowcover on Alexander Island, and the ITT example shows the development of proglacial lakes following glacier retreat on Snow Island in the South Shetland Islands. Though these four panel sets are designed to highlight the four main change classes, all change classes can be seen within these panels. Modern images are derived from Landsat 8 OLI, and the old images are derived from Landsat 7 ETM+.

We can also visually inspect the classes of change by looking at the map of change relative to real changes in the landscape viewed from satellite images (Figure 10). We can see that our change detection is good at detecting phase changes, such as melting ice (ITF and ITT); in the case of Alexander Island, this highlights the exposure of new sediments, whereas on Snow Island (Byers Peninsula site) this highlights the formation of new proglacial lakes. We are also able to detect more subtle changes in the flow of streams and the presence of wet sediments on James Ross Island (increased river activity, shown by FTC) and Seymour Island (James Ross Archipelago site) with reduced river activity and possible dust deposits.

### Data availability

The data used to produce these results, alongside the sampling points for the accuracy assessment and the spatial map of confidence, are available as TIFs and shapefiles from Stringer (2022). The land cover change maps produced from this paper are available from Stringer (2025).

Land class spectra are available in the supplementary materials.

## Discussion of study approach and limitations

### Methodological approach

#### Landcover classification

There is a dearth of available data with which to produce an independent training data set necessary for a supervised classification approach (e.g., random forest classification, support vector machine) for a wide-scale land classification in Antarctica (Rodriguez-Galiano et al. 2012). Therefore, we decided to use an unsupervised classification approach. Unsupervised approaches do not require training data sets and instead use the spectral characteristics of each pixel to statistically cluster similar pixels together without user input. The *K*-means algorithm is fully objective and removes the potential to target predefined classes that may be difficult to identify in medium-scale resolution satellite images or that may be in abundance in those areas visited by mapped areas (i.e., those producing training data) but not more widely (Grimes et al. 2024). This approach is particularly useful for large, national/regional-scale spatial analysis and has recently been applied to the classification of Greenland (Mohd Hasmadi, Pakhriazad, and Shahrin 2009; Grimes et al. 2024). Given that land cover data are disparate and incomplete over the study sites, this approach had the added benefit that our field knowledge, as well as information from published maps of relatively small areas (Table 1), could be used to interpret clusters that cover much wider areas.

#### Change detection

There are several ways in which change detection can be conducted, and these methods have previously been the subject of comprehensive literature reviews (Lu et al. 2004; Tewkesbury et al. 2015). The most commonly used of these techniques is postclassification comparisons of image pairs. This technique involves creating a land cover classification of images in two time periods and then directly comparing the change in classes. Although this method is intuitive, it is flawed because its overall accuracy is reliant on the accuracy of the two land cover products. Individual errors in each land cover map are compounded in the final map of change, resulting in unacceptably high uncertainty values (Lu et al. 2004; Tewkesbury et al. 2015). Change vector analysis determines the changes in the spectral properties of images over time, which allows for a classification that allows the specific type of change to be identified (Bovolo and Bruzzone 2007). Though CVA, as used in this study, has been criticized for being difficult to interpret (Carvalho Júnior et al. 2011), recent advances in this methodology mean that the method has increased the usability of the technique, as well as its ability to identify different types of change (Xu et al. 2018). CVA determines changes in the spectral properties of images over time and has the benefit of avoiding compounding errors (Lu et al. 2004; Tewkesbury et al. 2015).

### Study challenges and limitations

#### Land classification challenges

Previous studies have highlighted three key challenges when it comes to classifying terrestrial landcover: (1) distinguishing moisture levels in soils/sediments, (2) distinguishing sediment grain size, and (3) the spectral heterogeneity of bedrock. Though we have described land classes that use these terms, because they are useful geomorphological descriptors, we do not argue that we have solved these fundamental challenges associated with distinguishing between these groups spectrally but instead address how our study has arrived at its final classification scheme for these groups.

In terms of moisture, our coarse/wet sediment class came from clustering of mapped features such as scree slopes and braidplains. Previous research has shown that areas of scree slope and moisture sediments are typically associated with lower albedo values (Clark 1999; M. R. Salvatore et al. 2023), which likely accounts for why these groups were clustered together. Nonetheless, combining these two land types in a single class provides a useful indicator of geomorphologically active regions of the landscape.

In our study we found two challenges in classifying bedrock. The first of these challenges was associated with how bedrock is typically mapped versus how we have classified it. For example, in the Dry Valleys, bedrock accounts for 13 percent of the area and the performance of the classification is particularly notable for its ability to pick out an exposed basement sill (Petford and Mirhadizadeh 2017) in Wright Valley. In other studies (e.g., Jennings et al. 2021), bedrock classes are often overrepresented (Figure 4) because the study aims to map geomorphology or geology, rather than surface characteristics such as physical weathering and in situ production of block fields. Moreover, field observations show that boulders and other glacigenic sediments overlie many of the large igneous extrusions. Therefore, our classification gives a sense of mostly thin surface coverage of exposed solid bedrock. Previous work (e.g., M. R. Salvatore et al. 2014) has highlighted the spectral differences in different types of bedrock; indeed, we also found that several distinct clusters formed during our classification process that highlighted distinct igneous and metamorphic outcrops. For simplicity, these clusters were combined into a single “bedrock” class.

#### Study limitations and future work

Though we made every effort to minimize the differences in the time of year between image pairs and took further steps to ensure that there was evidence of hydrological activity and minimal snow cover, there remains the possibility that some of the changes we detected were due to a differences in growing season or hydrological season or unusual weather events. In particular, those seasonal factors could affect the area of the vegetation and coarse/wet sediment classes. Future studies should seek to ensure that ground conditions are similar when conducting change detection, the first step of which is to ensure that images are from as close to the same part of the hydrological and growing season as possible. Though it is possible to distinguish between glacial ice and snow (e.g., Awasthi and Varade 2021; Li, Wang, and Wu 2022), many previous land classifications of polar regions have not done so (Wang et al. 2020; Grimes et al. 2024). Some recent studies have made use of snow masking algorithms (e.g., Roland et al. 2024); however, this in itself presents a challenge in that it can alter the land area compared during change detection, which introduces further uncertainty. Therefore, we took the decision to follow the tried-and-tested approach of choosing images with limited visible snow cover.

One of the key challenges of any remote sensing study is validation, and this has been the topic of considerable discussion and review (e.g., Olofsson et al. 2013, 2014). A difficulty found in our study was the lack of existing data sets with which to validate our approach. Furthermore, those independent data sets that do exist were already exploited to aid us in the interpretation of *K*-means clusters (Table 1). Therefore, similar to previous research in remote regions (Grimes et al. 2024), we used our interpretations of higher resolution satellite imagery for validation. This may have introduced some biases through the misclassification of validation points, but we contend that this was preferable to introducing biases from validating our approach against data sets that were used in the initial classification process. The challenges in validating this work highlight the need for further mapping of Antarctic regions based on field observations. Alexander Island, in particular, was difficult to classify due to a lack of supporting material to aid our cluster interpretations; the most recent geological map is from 1981 (British Antarctic Survey 1981), and only limited geomorphological maps of the region exist (M. C. Salvatore 2001). This site highlights the need to collect more high-quality ground data in Antarctica to improve our wider understanding of proglacial environments in the southernmost continent. Even projects to produce high-quality maps in small areas of these remote regions would improve the performance of remote techniques, such as those described in this study.

## Summary and conclusions

In this study, we have created a land cover map of the major proglacial regions of sub-Antarctic islands, the Antarctic Peninsula Region, and the McMurdo Dry Valleys. Given the lack of consistent land cover or geomorphology maps in Antarctica, we used an unsupervised *K*-means clustering approach to classify 30-m-resolution Landsat 8 OLI images by interpreting clusters in a hierarchical approach using our expert judgment and field experience in Antarctica. We present information on the coverage of nine land cover classes: turbid water, water, wet ice, ice, land (nondifferentiated), bedrock, fine sediment, coarse sediment, and vegetation. We mapped eight distinct land surface (plus a no data and land [undifferentiated] class) at 30 m, with an accuracy of 77.0 percent for proglacial classes and 92.2 percent for ice. We also highlighted the spatial pattern in land classes, notably in vegetation and coarse/wet sediment, which are typically more abundant in sites that are more northerly.

Additionally, we analyzed land cover changes in the proglacial regions of Antarctica, which we achieved using a CVA approach at an accuracy of 80.1 percent. Through our analysis of change, we highlighted a latitudinal pattern in ice loss; the proportion of landscape change on South Georgia due to the loss of ice is two orders of magnitude greater than that in the Dry Valleys. This change also occurs in tandem with the opposite pattern occurring in the sediment class changes; this is possibly also influenced by an increase in vegetation coverage in more northern sites. We also highlighted the extensive change in the landscape that has occurred on Alexander Island, where 50 percent of the proglacial coverage has changed this century, likely as a consequence of recent dramatic warming events around the George VI ice shelf.

This data set provides a first step in understanding the makeup of Antarctica’s important proglacial regions. It also highlights the need for greater ground-verified data to improve the accuracy of future Antarctic land classifications. We expect that these data will further research in several disciplines, particularly those that focus on ecology, environmental sciences, and atmospheric sciences, and will provide an important first data set for monitoring environmental and ecological change in Antarctica.

## Supplementary Material

Spectra_toSubmit.xlsx

Supplementary.docx

## Code availability

Stringer, C.D. (2022). Contemporary (2016–2020) land cover across West Antarctica and the McMurdo Dry Valleys [Code] (Version 1). Zenodo. https://doi.org/10.5281/zenodo.6720051

Stringer, C.D. (2023). 21st century land cover change across the major proglacial regions of West Antarctica and the McMurdo Dry Valleys [Code]. (Version v1). Zenodo. https://doi.org/10.5281/zenodo.7991208
