# Supplementary material for: Land cover change across the major proglacial regions of the sub-Antarctic islands, Antarctic Peninsula, and McMurdo Dry Valleys, during the 21st century
Source: Arct Antarct Alp Res. 2025 May 7;57(1):2483474. doi: 10.1080/15230430.2025.2483474 (PMC12311964; doi:10.1080/15230430.2025.2483474)
Supplement: Supplementary.docx [file UAAR_A_2483474_SM6901.docx]

**1. Methods**

**1.1. Choice of K-means values**

To produce our land classification map, we used a K-means clustering algorithm to split each image into 75 (K value = 75) discrete clusters. Unsupervised approaches, such as K-means do not require training datasets, and instead use the structure of an image to identify spectrally homogeneous pixels, based on a user-defined number of clusters; this is particularly useful for sites with little field information (Duda and Canty, 2002; Mohd Hasmadi et al., 2009), such as those analysed in this study.

The specific K values were determined through expert judgement and represent values that minimised the chance of misclassification. Whilst others have used statistical methods, such as the ‘Elbow Method’, to determine the number of clusters for analysis (Syakur et al., 2018), we chose to use our expert judgement because it allowed us to find a suitable threshold to properly identify the different land cover classes as independently mapped (Table 1) and identified in the field. The K value chosen ultimately affects the accuracy of the output (Ahmed et al., 2020) and it is, therefore, essential to assess the accuracy of the final product using independent datasets. The clusters are determined using the spectral information of each image, based on 500,000 randomly selected sampling points. We assigned each of these sections a first-order class by visually inspecting the image they were derived from. In some cases, we could not easily assign a cluster a first-order class. This was usually because a cluster had conflated shadow with dark seawater. To address this, we split these clusters using a slope threshold of 3°, with pixels <3° being assigned as water. Where this process resulted in obvious misclassification we used a random forest classifier to differentiate between water, land and ice. Some pixels were covered entirely by very dark shadows or clouds and, therefore, we could not classify them; these were assigned “No data”.

**1.2. No data/land undifferentiated classes**

The largest of these examples are on South Georgia and James Ross Island. To the northwest of South Georgia (Cape Alexandra and Bird Island), we classified a large area of land as “no data”, since it was entirely obscured by thick clouds in images. Similarly, we classified the southeast of James Ross Island (the largest island in the James Ross Archipelago) as “Land (undifferentiated)”. This region was covered by thin clouds in the imagery, which allowed us to differentiate land from ice and water, but it meant that we could not assign the land a second-order class with any confidence.

**1.3. Use of maps to classify K-means clusters**

Each map used its own nomenclature, but we found different land classes primarily centred on vegetation, bedrock outcrops, and landforms made of unlithified sedimentary rocks that are often defined by their grain size. Of the proglacial land classes, the two sedimentary classes (coarse/wet sediment and fine and dry sediment) are dominant (73 % - 90 % coverage).

**1.4. Change detection**

First, we merged each image pair to create an 18-band image with spectral information from both images (i.e. Band1_L7_, Band1_L8_, Band2_L7_, Band2_L8_ … ). We then added three further bands to describe: **i)** the magnitude of change in reflectance intensity between the images in each image pair, as described by the Euclidian distance (ED, Eq. (4)); **ii)** the change vector direction angle (DA. Eq. (5)); and, **iii)** the spectral angle mapper (SAM, Eq. (6)).

$ED= \sqrt{\sum_{i=1}^{n} d_{i}^{2}}, ED \in[ 0, max (ED)]$ (4)

$DA= \cos^{-1} \left[ \frac{\sum_{i}^{n} d_{i}}{\sqrt{n}*ED} \right], \alpha\in[ 0, \pi]$ (5)

$SAM= \cos^{-1} \left[ \frac{\sum_{i}^{n} Y_{i}* X_{i}}{\left\| Y_{i} \right\|\left\| X_{i} \right\|} \right]$ (6)

Where:

- $d_{i}$ is the difference in values for each spectral pair.
- *X_i_*  represent the spectral information of the first image
- *Y_i_*  represent the spectral information of the second image
- ‖ ‖ represents the length of each vector

This 21-band image was then classified via a training dataset. To produce a training data set we classified the Landsat 7 image of Byers’ Peninsula using the approach laid out in section 2.2.2 (i.e. K-means). We chose this site because it had the greatest variety of land classes in the contemporary classification. Across this site, we randomly selected 8,500 points and extracted the land cover at each point from both time-periods and assigned each a class-to-class (ClTCl) change value based on their land cover classification in the Landsat 7 (L7) image and Landsat 8 image (i.e. L7TL8). We removed any ClTCl changes that represented less than 1 % of the points to reduce the risk of misclassification. The remaining points described 12 ClTCl change classes (Table 2).

We then extracted band values from the 21-band image at each of these points and used them to train a random forest classifier that classified change at each site. The classifier was parameterised to have 500 trees because errors are stable around this number (Lawrence et al., 2006; Xu et al., 2018) and used to classify the 21 band image at each site. We modified the training dataset for each of our five proglacial sites to ensure that only changes between classes present in the modern land classification were possible.. As well as representing an absolute change in land cover type, change classes also describe processes. For example, the CTT class both describes a change from coarse sediment to turbid water, as well as representing a change from land to water. In the case of Alexander Island, there is no coarse sediment land cover or turbid water in either land classification. However, the CVA identified some pixels of CTT change. Therefore, we did not remove CTT as a possible change class as it accurately identified a process that was clearly visible in satellite images (i.e. ponded water where land previously was).

**1.5 Confusion matrices**

**Confusion matrix for all land cover classes**

**Confusion matrix of proglacial classes**

**1.6 Images used during the analysis, including the date of image acquisition and overall cloud cover**

| **Landsat 8 image** | **Date** | **Cloud cover %** | **Landsat 7 image** | **Date** | **Cloud cover %** |  |
| --- | --- | --- | --- | --- | --- | --- |
| **James Ross Island** | | | | | | |
| LANDSAT/LC08/C02/T2_TOA/LC08_215105_20170204  LANDSAT/LC08/C02/T2_TOA/LC08_215105_20160202 | 04/02/2017  02/02/2016 | 6  6 | LANDSAT/LE07/C02/T2_TOA/LE07_216105_20000221 | 21/02/2000 | 15 |  |
| **Dry Valleys** | | | | | | |
| LANDSAT/LC08/C02/T2_TOA/LC08_056116_20191217 | 17/12/2019 | 0 | LANDSAT/LE07/C02/T2_TOA/LE07_059115_20011228 | 28/12/2000 | 1 |  |
| **Alexander Island** | | | | | | |
| LANDSAT/LC08/C02/T2_TOA/LC08_218110_20200117  LANDSAT/LC08/C02/T2_TOA/LC08_217111_20191107  LANDSAT/LC08/C02/T2_TOA/LC08_216110_20191218 | 17/01/2020  07/11/2019  18/12/2019 | 0  1  0 | LANDSAT/LE07/C02/T2_TOA/LE07_213111_20020104  LANDSAT/LE07/C02/T2_TOA/LE07_217111_20021202  LANDSAT/LE07/C02/T2_TOA/LE07_218110_20010104  LANDSAT/LE07/C02/T2_TOA/LE07_218111_20030211  LANDSAT/LE07/C02/T2_TOA/LE07_214110_20030130  LANDSAT/LE07/C02/T2_TOA/LE07_218110_20030211  LANDSAT/LE07/C02/T2_TOA/LE07_219109_20011229  LANDSAT/LE07/C02/T2_TOA/LE07_132133_20001123  LANDSAT/LE07/C02/T2_TOA/LE07_216111_20030112  LANDSAT/LE07/C02/T2_TOA/LE07_217111_20010214  LANDSAT/LE07/C02/T2_TOA/LE07_214110_20020127  LANDSAT/LE07/C02/T2_TOA/LE07_217110_20021202  LANDSAT/LE07/C02/T2_TOA/LE07_218111_20010104 | 04/01/2002  02/12/2002  04/01/2001  11/02/2003  30/01/2003  11/02/2003  29/12/2001  23/11/2000  12/01/2003  14/02/2001  27/01/2002  02/12/2002  04/01/2001 | 1  1  1  1  2  2  2  3  3  3  4  4  4 |  |
| **Deception Island** | | | | | | |
| LANDSAT/LC08/C02/T2_TOA/LC08_219104_20200209 | 09/02/2020 | 21 |  |  |  |  |
| **Byers Peninsula** | | | | | | |
| LANDSAT/LC08/C02/T2_TOA/LC08_219104_20200209 | 09/02/2020 | 21 | LANDSAT/LE07/C02/T2_TOA/LE07_219104_20020130 | 30/01/2002 | 17 |  |
| **South Georgia** | | | | | | |
| LANDSAT/LC08/C02/T1_TOA/LC08_206098_20180328  LANDSAT/LC08/C02/T1_TOA/LC08_207098_20180404 | 28/03/2018  04/04/2018 | 2  47 | LANDSAT/LE07/C02/T1_TOA/LE07_206098_20020103  LANDSAT/LE07/C02/T1_TOA/LE07_206098_20030207 | 03/01/2002  07/02/2003 | 65  17 |  |

**Images used in accuracy assessment**

Accuracy assessment was conducted using Sentinel-2 MSI images that coincided with the date of image acquisition of the Landsat-8 OLI images used for the land class classification. Images with low-cloud images preferentially chosen. The code to collate these images, as well as list of images (in the console) can be found here: <https://code.earthengine.google.com/6bc925765ad1a42d193d2ef43930f483> . NB: image availability was prioritised over cloud-free images. Any validation point located over cloud cover was discounted from the final accuracy assessment.

**1.7 Mean spectra**

**Spectra:**


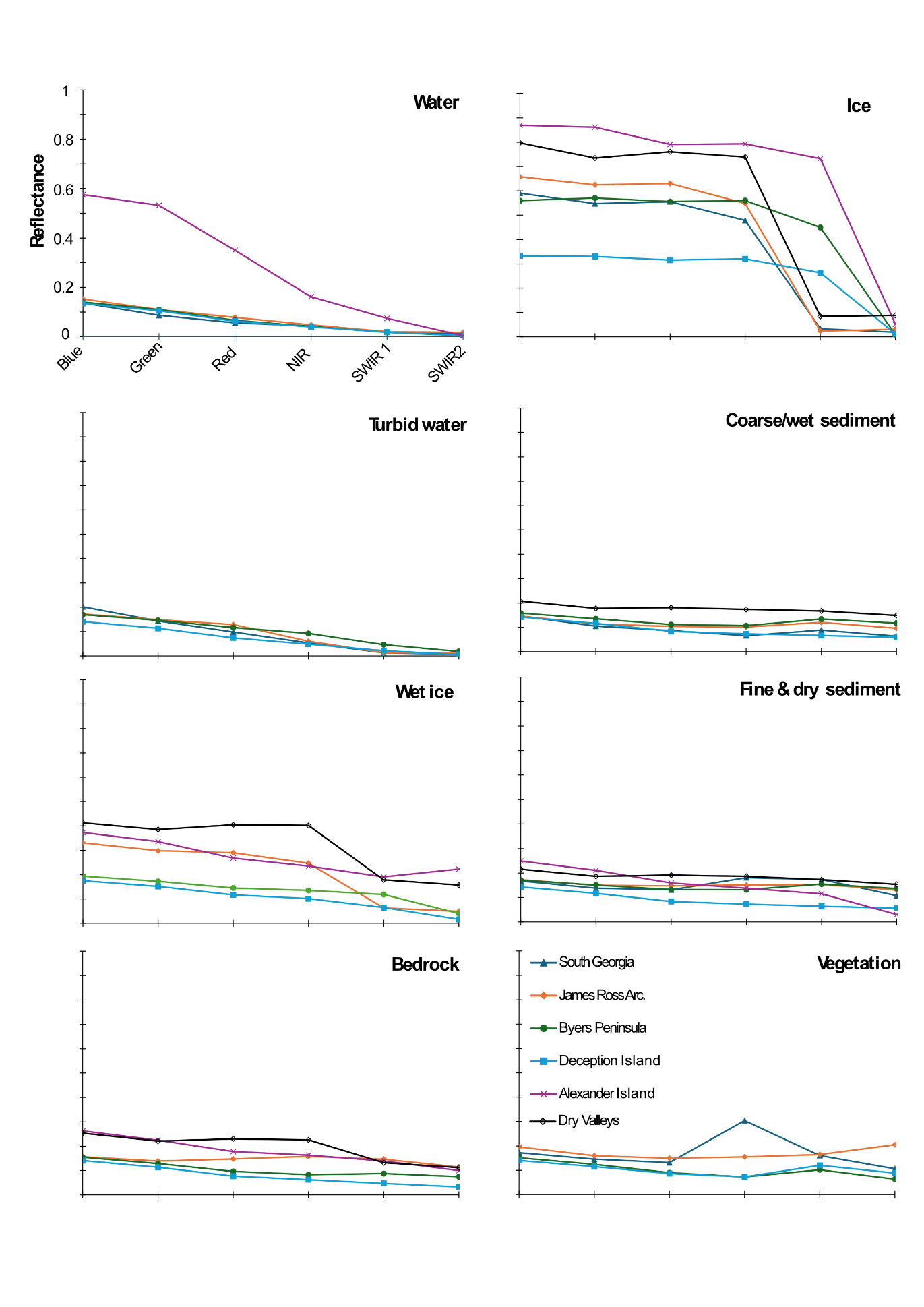


**Comparisons:**

NB: dotted line = coarse/turbid


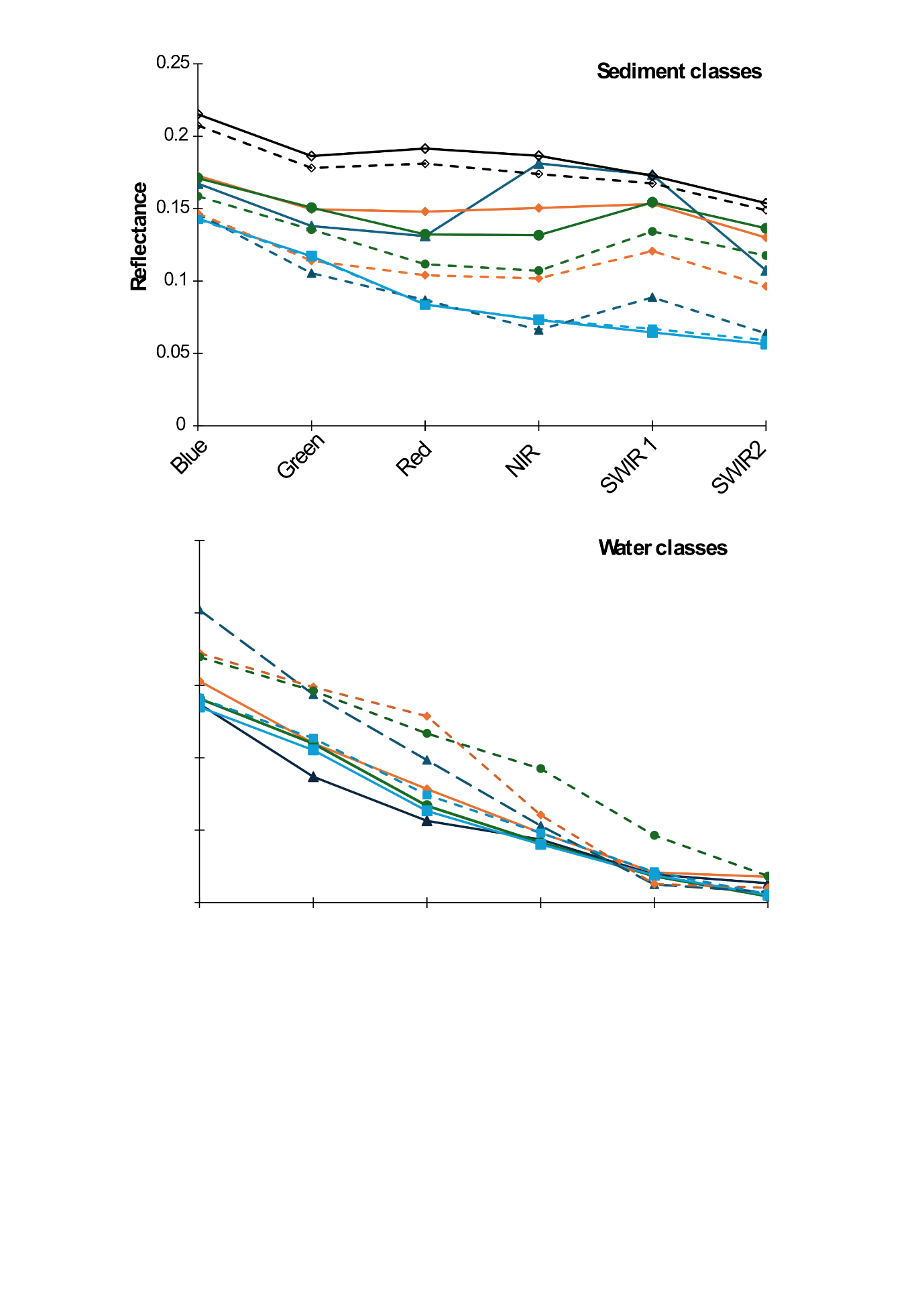


**
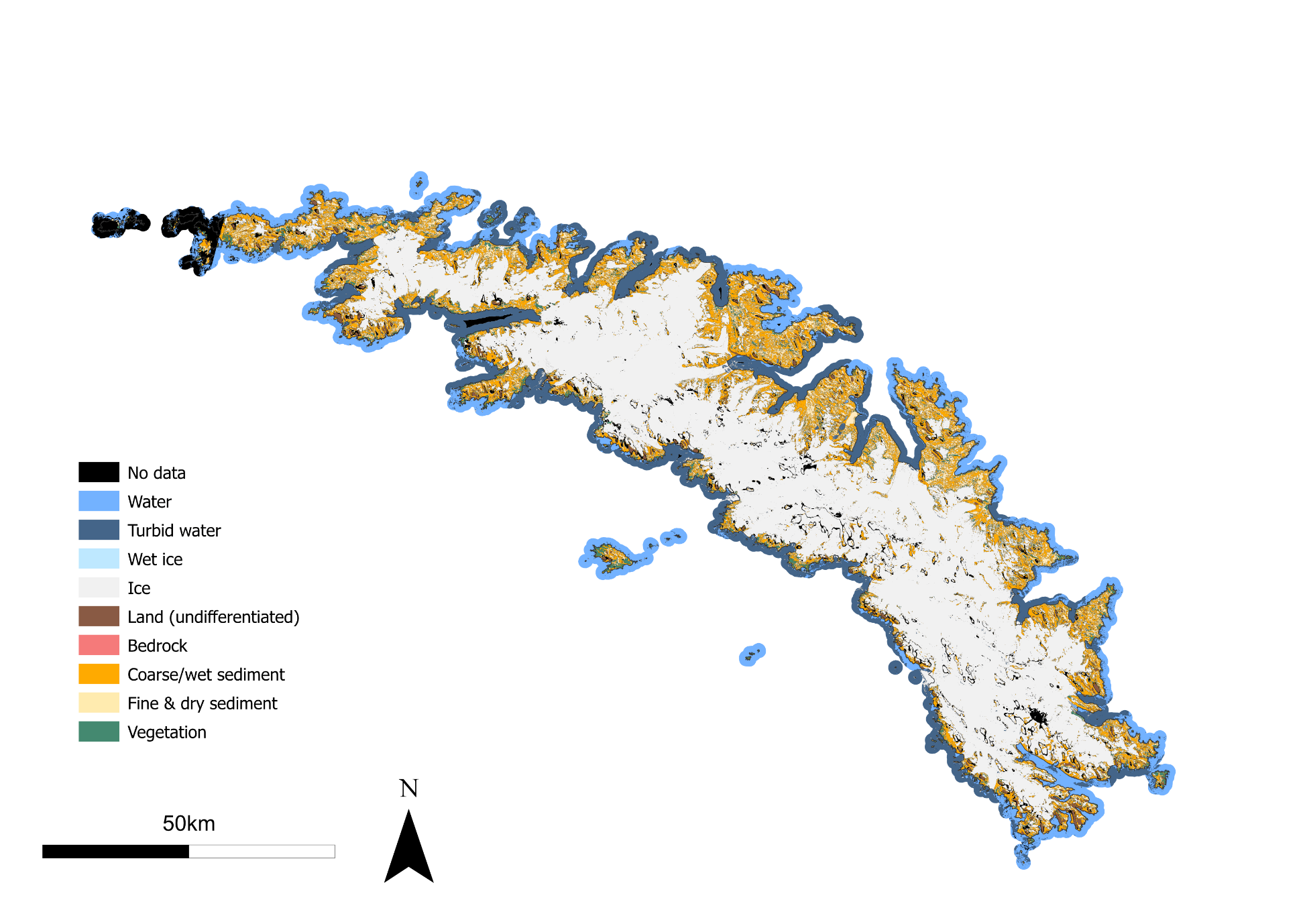
2. Land classification maps**

**South Georgia**

**
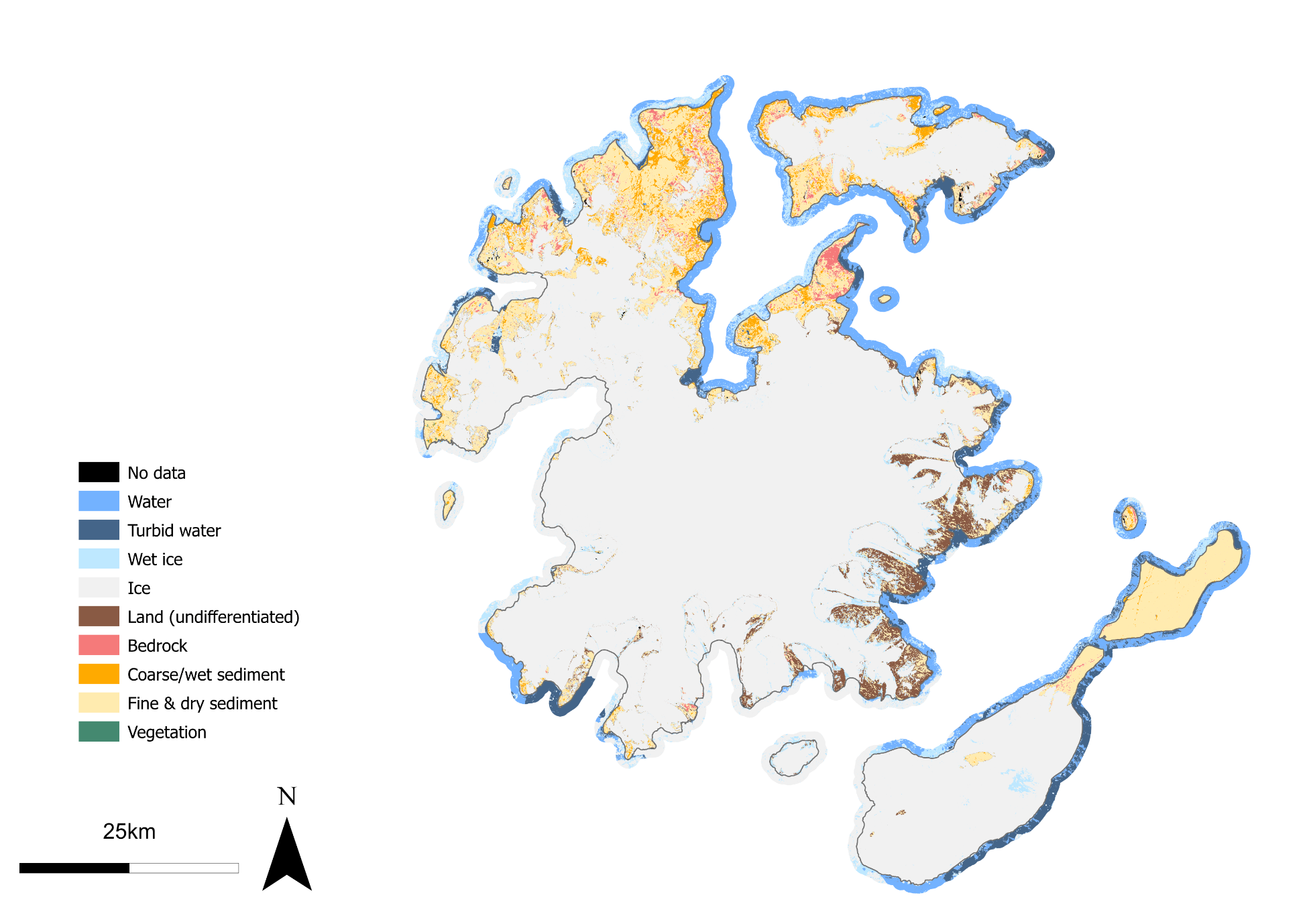
James Ross Archipelago**

**Byers Peninsula**

**
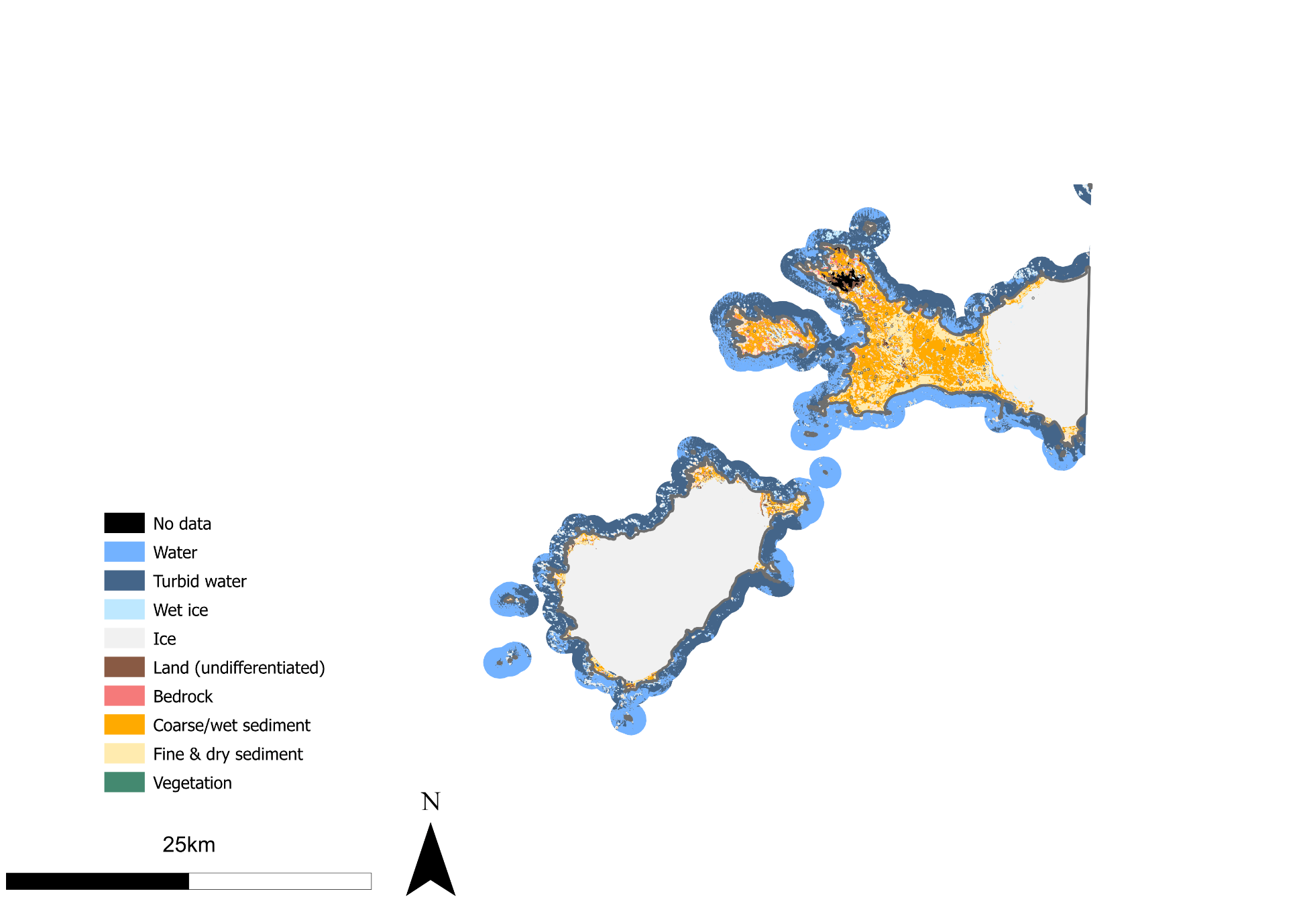
**

**
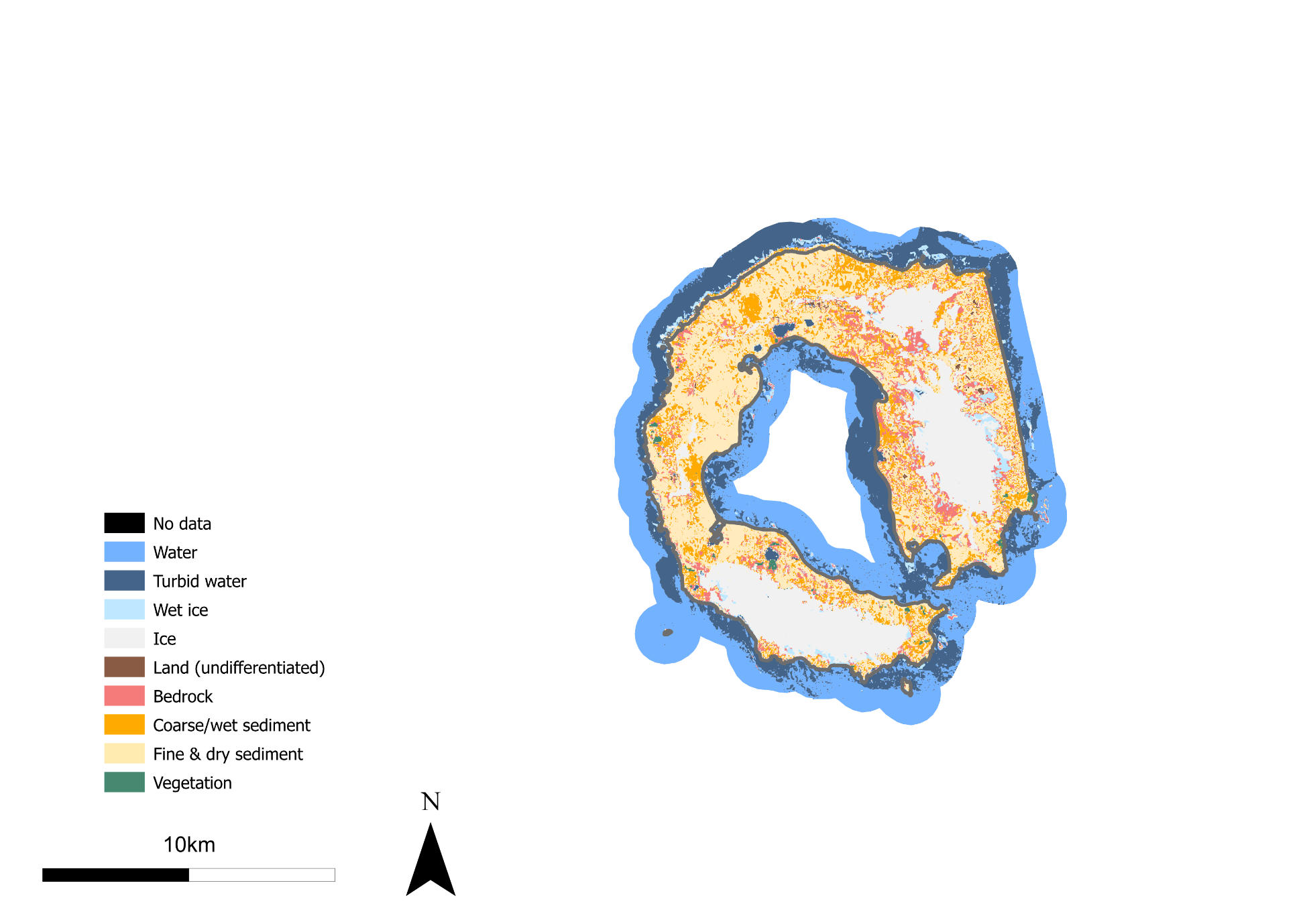
Deception Island**

**Alexander Island**

**
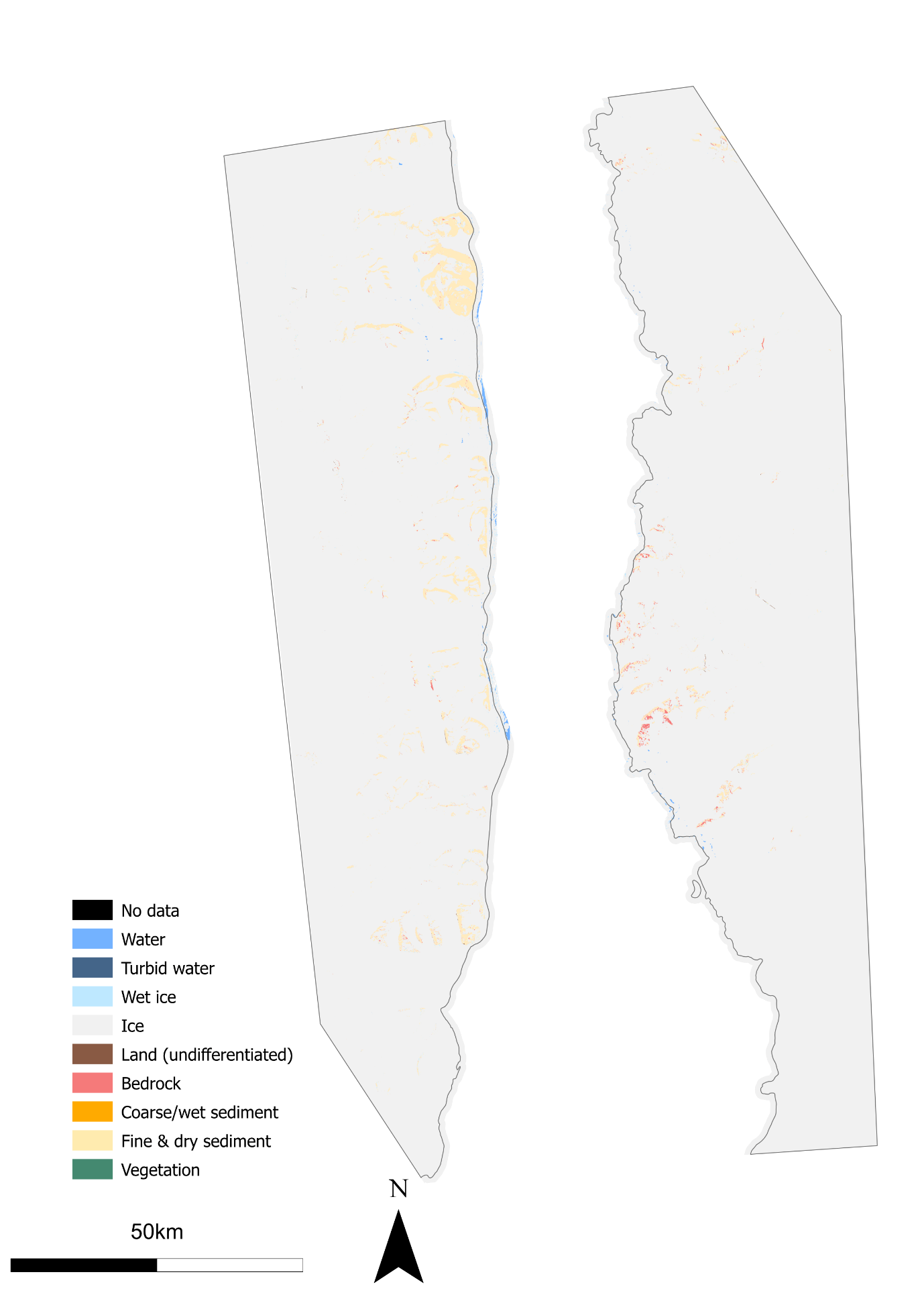
**

**
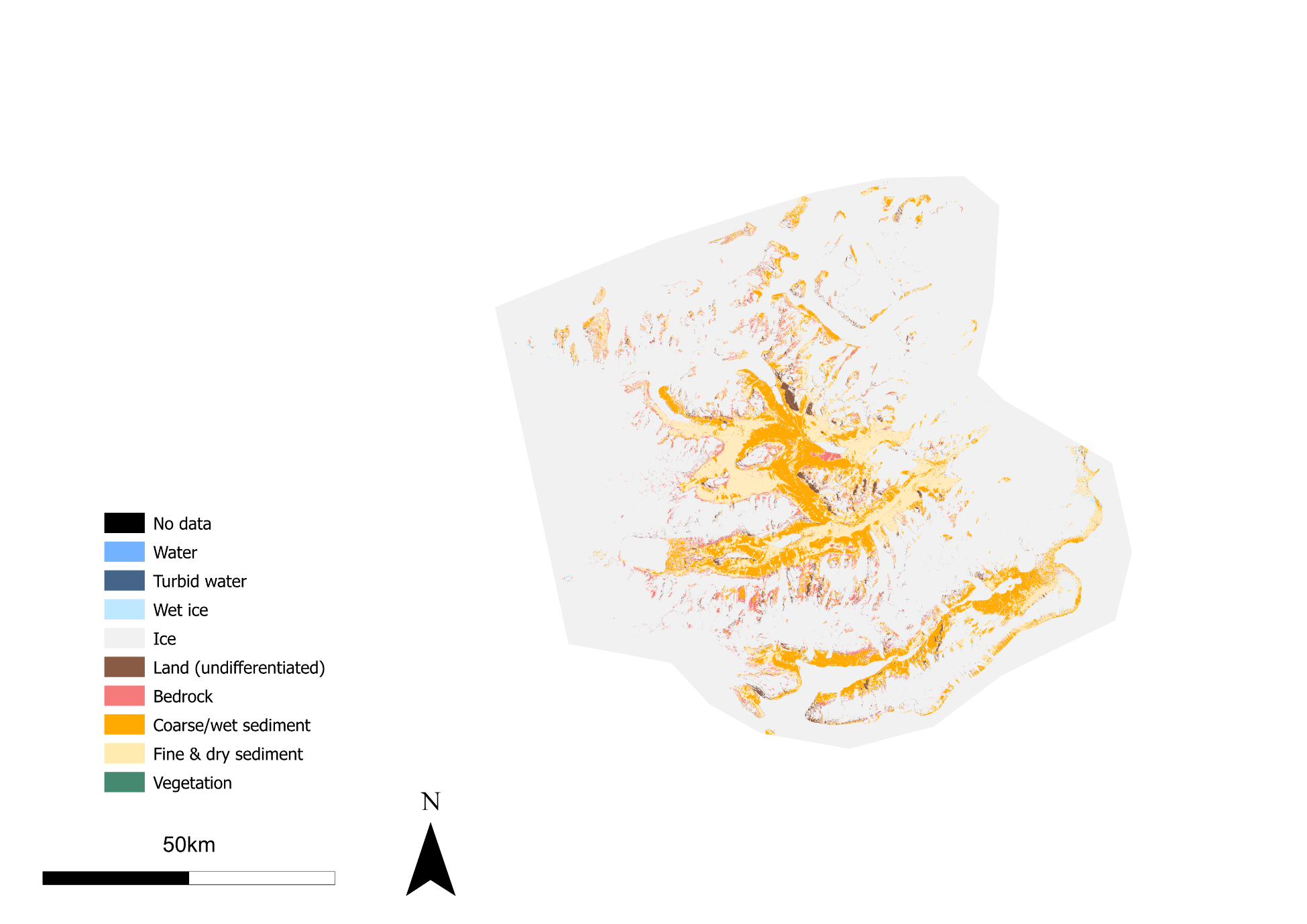
Dry Valleys**

**References**

Ahmed, M., Seraj, R. and Islam, S.M.S. 2020. The k-means algorithm: A comprehensive survey and performance evaluation. *Electronics (Switzerland)*. **9**(8), pp.1–12.

Duda, T. and Canty, M. 2002. Unsupervised classification of satellite imagery: Choosing a good algorithm. *International Journal of Remote Sensing*. **23**(11), pp.2193–2212.

Mohd Hasmadi, I., Pakhriazad, H.Z. and Shahrin, M.F. 2009. Evaluating supervised and unsupervised techniques for land cover mapping using remote sensing data. *Malaysia nJournal of Society and Space*. **5**(1), pp.1–10.

Syakur, M.A., Khotimah, B.K., Rochman, E.M.S. and Satoto, B.D. 2018. Integration K-Means Clustering Method and Elbow Method For Identification of The Best Customer Profile Cluster. *IOP Conference Series: Materials Science and Engineering*. **336**(1), p.12017.
